# Spire and Formin 2 Synergize and Antagonize in Regulating Actin Assembly in Meiosis by a Ping-Pong Mechanism

**DOI:** 10.1371/journal.pbio.1001795

**Published:** 2014-02-25

**Authors:** Pierre Montaville, Antoine Jégou, Julien Pernier, Christel Compper, Bérengère Guichard, Binyam Mogessie, Melina Schuh, Guillaume Romet-Lemonne, Marie-France Carlier

**Affiliations:** 1 Laboratoire d'Enzymologie et Biochimie Structurale, CNRS, Gif-sur-Yvette, France; 2 MRC Laboratory of Molecular Biology, Cambridge, United Kingdom; Beatson Institute, United Kingdom

## Abstract

In mammalian oocytes, three actin binding proteins, Formin 2 (Fmn2), Spire, and profilin, synergistically organize a dynamic cytoplasmic actin meshwork that mediates translocation of the spindle toward the cortex and is required for successful fertilization. Here we characterize Fmn2 and elucidate the molecular mechanism for this synergy, using bulk solution and individual filament kinetic measurements of actin assembly dynamics. We show that by capping filament barbed ends, Spire recruits Fmn2 and facilitates its association with barbed ends, followed by rapid processive assembly and release of Spire. In the presence of actin, profilin, Spire, and Fmn2, filaments display alternating phases of rapid processive assembly and arrested growth, driven by a “ping-pong” mechanism, in which Spire and Fmn2 alternately kick off each other from the barbed ends. The results are validated by the effects of injection of Spire, Fmn2, and their interacting moieties in mouse oocytes. This original mechanism of regulation of a Rho-GTPase–independent formin, recruited by Spire at Rab11a-positive vesicles, supports a model for modulation of a dynamic actin-vesicle meshwork in the oocyte at the origin of asymmetric positioning of the meiotic spindle.

## Abstract 2

An in vitro study reveals how the three actin binding proteins profilin, formin 2, and Spire functionally cooperate by a ping-pong mechanism to regulate actin assembly during reproductive cell division.

## Author Summary

Mammalian reproduction requires successful meiosis, which consists of two strongly asymmetric cell divisions. In meiosis I, movement of the spindle (the subcellular structure that segregates chromosomes during division) toward the oocyte cortex (the outer layer of the egg) is essential for fertility. This process requires that actin filaments assemble in a dynamic mesh, driven by three actin binding proteins, profilin, formin 2, and Spire. To date the molecular mechanisms by which these three proteins cooperate are not known. We now explore this *in vitro* by a combination of bulk solution and single actin filament assembly assays in the presence of profilin, Spire, and formin 2. Individually, Spire binds to actin filament ends to block their growth, and by itself, formin 2 associates poorly with filament ends, promoting fast processive assembly from the profilin-actin complex. However, when present together, Spire and formin 2 interact with one another (the formin 2 C-terminal binds to the N terminal Spire KIND domain), forming transient complexes at filament ends from which each binds alternately to the filament ends to regulate actin assembly by a ping-pong mechanism. Our *in vitro* observations are validated by injection studies in mouse oocytes. In oocytes, the additional interaction of Spire and formin 2 with Rab11a-myosin Vb vesicles couples high actin dynamics to vesicle traffic.

## Introduction

In mouse meiosis I, translocation of the spindle toward a cortical site that defines polar body extrusion is the first step in establishment of oocyte polarity [1],[2]. This process is driven by assembly of cytoplasmic actin filaments in which formin 2 (Fmn2) plays a pivotal role [3]–[8]. Loss of Fmn2 prevents correct positioning of the metaphase spindle and causes pregnancy loss and infertility [9]. The mechanism of actin-based translocation of the spindle is an important issue in cell biology [7]. Fmn2 is required for assembly of an isotropic, dynamic cytoplasmic network, but the mechanism by which actin assembly drives asymmetric spindle positioning is not understood [3],[7],[10]. Local myosin-dependent pulling on the actin meshwork in the spindle pole region has been proposed [5],[11]. Other studies suggest that the spindle is pushed by Fmn2-induced insertional assembly of filaments around the spindle [4]. Other actin-based mechanisms seem posssible considering the very slow rate of spindle translocation. A recent report indicates that in mouse oocytes, actin nucleators are clustered on Rab11a-positive vesicles associated with myosin Vb and that Rab11a and myosin Vb are also required for asymmetric positioning [12].

Fmn2 cooperates with two other actin binding proteins, Spire and profilin. Genetic interactions between Spire, formin Cappuccino (the ortholog of Fmn2 in *Drosophila*), and profilin were first revealed in polarity axis patterning of the *Drosophila* oocyte [13]–[15]. In the mouse oocyte, overexpression studies suggest that Spire and Fmn2 cooperate in a functional unit to achieve spindle translocation [6]. Fmn2 and Spire also display nearly identical expression patterns in developing and adult nervous tissues [16].

Fmn2 and Cappucccino are members of the Fmn family of Rho-GTPase–independent formins. The autoregulatory DAD domain of Diaphanous-related formins (DRFs) is replaced by a short FH2 tail sequence that makes an inhibitory contact with the N-terminal region in Cappuccino [17].

Spire is a modular protein. The N-terminal region (Nt-Spire) consists of a kinase-like noncatalytic domain (KIND) followed by four consecutive WH2 domains that bind actin. The C-terminal moiety contains a Spir box and a FYVE-related domain, potentially interacting with Rab GTPases and membranes [18]. Nt-Spire nucleates actin assembly *in vitro* in the absence of profilin [19]. Under physiological conditions where profilin-actin (PA) complex is the main form of polymerizable actin, the binding of Nt-Spire to filament barbed ends blocks assembly from PA [20],[21].

Spire and Fmn2 directly interact [22] via association of the C-terminal tail of the FH2 domain of Fmn2/Capu with the KIND domain of Spire [23]–[26]. Binding of KIND to the isolated FH2 domain of Cappuccino inhibits FH2-induced stimulation of actin assembly [23],[25].

The synergy observed *in vivo* between Spire and Fmn2 contrasts with *in vitro* evidence for opposite effects of Nt-Spire and Fmn2, taken individually, on filament barbed end assembly and for the inhibition of FH2 by KIND. To understand the molecular mechanism by which Spire and Fmn2 act in synergy to promote actin assembly and spindle translocation, here we perform bulk solution and single filament assays of the interplay between Nt-Spire, Fmn2, and profilin in actin assembly. We find that Nt-Spire binding to barbed ends facilitates the recruitment of Fmn2 via direct interaction between the KIND domain of Spire and the C-terminal region of Fmn2, called Formin-Spire Interacting (FSI) region, followed by release of Nt-Spire and fast processive filament growth. In the presence of Nt-Spire, Fmn2, and PA, filaments display rapid processive growth interrupted by pauses due to the alternating barbed end occupancy by Fmn2 and Nt-Spire, acting in an original “ping-pong” mechanism. *In vitro* data, validated by the effects of injected proteins in the mouse oocyte, lead to a comprehensive model of coupled dynamics of actin filaments and Rab11a vesicles.

## Results

### Fmn2-Induced Filament Assembly from PA Is Inhibited by the Isolated KIND Domain, but Stimulated by Nt-Spire

**Figure 1:**
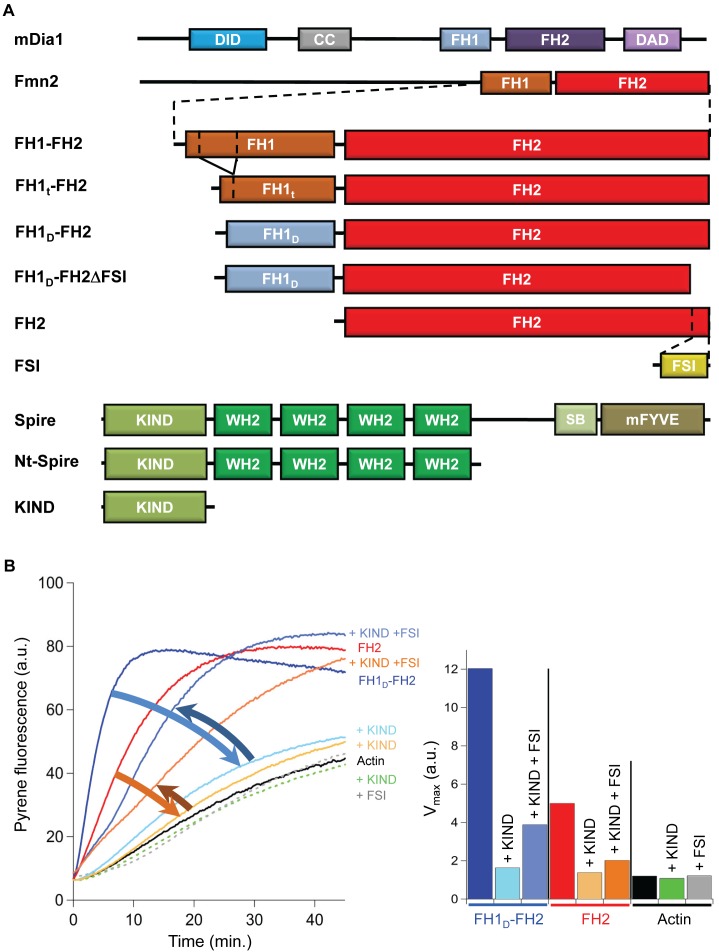
Spontaneous assembly of actin is stimulated by FH2 or FH1_D_-FH2 and its inhibition by the KIND domain of Nt-Spire is relieved by FSI. (A) Structural organization of Fmn2 and Spire and schematics of the constructs used. Vertical dashed lines in the FH1 domain of the FH1-FH2 construct delineate the truncated region of the FH1t-FH2 construct. FH1_D_-FH2 represents the chimera comprising the FH1 domain of mDia1 and the FH2 domain of Fmn2. The 20 last C-terminal residues (FSI peptide) are deleted in FH1_D_-FH2ΔFSI. (B) Assembly of actin (2.5 µM, 10% pyrenyl-labeled, black line) is not affected by either KIND (0.5 µM, green dotted line) or FSI (2.8 µM, grey dotted line). Stimulation of actin assembly by 50 nM FH2 or FH1_D_-FH2, inhibition of the stimulation by KIND, and its restoration by FSI are color coded in red-orange for FH2 and purple-blue for FH1_D_-FH2. Histograms represent the change in global rate of spontaneous assembly (same color coding).

We purified constructs of human Nt-Spire comprising the N-terminal KIND domain and the 4 WH2 domains, of the isolated KIND domain, of the FH2 and FH1-FH2 domains of mouse Fmn2, and the more soluble truncated FH1_t_-FH2 and mDia1-chimeric FH1_D_-FH2 (Figure 1A, Materials and Methods). The FH2 includes the C-terminal region of interaction with KIND, called “tail” or “FSI.” A FSI-deleted construct FH1_D_-FH2ΔFSI was purified as well. The FSI peptide was chemically synthesized. As demonstrated along the paper, FH1_t_-FH2 and FH1_D_-FH2 behaved quantitatively identical to the original FH1-FH2 domain of Fmn2. This result indicates that the original FH2 domain of Fmn2, but not the nature and proline content of the FH1 domain, is essential in the activity and regulation of formin 2 by Spire. Most quantitatively detailed data were collected with FH1_D_-FH2. We further checked that all main properties resulting from interactions between Nt-Spire and FH1_D_-FH2, were reproduced with FH1-FH2 of Fmn2.

FH1_D_-FH2 stimulates filament assembly from MgATP-G-actin (in the absence of profilin) more efficiently than the isolated FH2 domain (Figure 1B). The FSI peptide did not affect assembly of actin alone, nor Nt-Spire-nucleated actin assembly, in contrast with a previous report [23]. The isolated KIND domain did not affect assembly of actin alone but inhibited FH2- or FH1_D_-FH2-stimulated polymerization. The FSI peptide abrogated the inhibitory effect of KIND, as reported with the mammalian proteins and their *Drosophila* orthologs Dm-Spir-KIND, Capu-CT, and Capu tail [24],[25].

**Figure 2:**
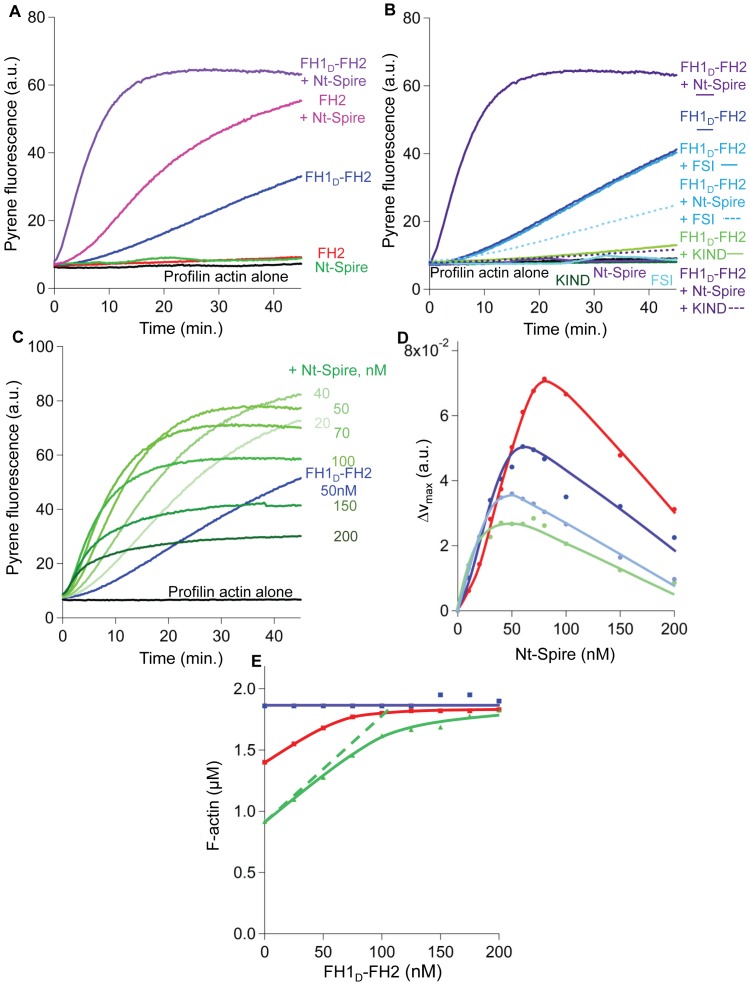
Fmn2 and Nt-Spire synergize to enhance filament assembly. (A) Filament spontaneous assembly from PA (2.5 µM actin, 6 µM profilin, black curve) in the presence of FH1_D_-FH2 (50 nM) or FH2 (50 nM) alone or with addition of Nt-Spire (50 nM). (B) KIND and FSI abolish the synergy between Nt-Spire and FH1_D_-FH2 in filament assembly. Conditions are as in (A), with 1.3 µM KIND and 2.8 µM FSI where indicated. Control curves in absence of KIND and FSI are duplicates of those in (A). (C) Concentration dependence of Nt-Spire in activation of filament assembly by FH1_D_-FH2. Conditions are as under (A), with color coded (light green to dark green) in order of increasing concentrations of Nt-Spire. (D) Nt-Spire and FH1_D_-FH2 interact with high affinity to stimulate assembly from PA. The maximal rate of actin assembly was measured after the lag, at 12.5 nM (green), 18 nM (light blue), 25 nM (dark blue), and 50 nM (red) FH1_D_-FH2, and increasing concentrations of Nt-Spire. (E) The amount of F-actin assembled at steady state in the presence of profilin is controlled by the relative amounts of FH1_D_-FH2 and Nt-Spire. Actin (2 µM, 2% pyrenyl labeled) was assembled at steady state in the presence of 2 µM profilin in the absence (purple curve) or presence of Nt-Spire (red curve, 50 nM; green curve, 100 nM), and FH1_D_-FH2 as indicated.

Profilin, the FH2 domain of formins, and Spire (via WH2 domains) all bind the barbed face of actin individually. The mutually exclusive binding of the three proteins to actin is at the heart of the puzzling mechanism by which they act in synergy. This issue was thus addressed in a straightforward fashion by monitoring spontaneous assembly of filaments from PA in the presence of either Fmn2, or Spire or both together. Profilin by itself strongly inhibits actin nucleation (Figure 2A, black line). FH1_D_-FH2 (Figure 2A, blue line), but not FH2 (Figure 2A, red line), promoted filament assembly from PA, like other formins [27]–[29], albeit much less efficiently. The FH1-FH2 of mDia1 showed the same nucleation activity at a one order of magnitude lower concentration (unpublished data). Nt-Spire did not support assembly from PA (Figure 2A, green line), consistent with the known capping of barbed ends by Nt-Spire [20]. In this experiment (2.5 µM actin, 6 µM profilin) the concentration of PA is 2.44 µM, and 0.06 µM actin is unliganded. Since no nucleation was observed, in the absence of FH1_D_-FH2 or in presence of FH2 only, over at least 1 h, and since FH1_D_-FH2 does not nucleate assembly of 0.06 µM actin, we conclude that FH1_D_-FH2 most likely nucleates and assembles filaments from PA.

Remarkably, in this physiological situation, where PA is the polymerizing form of actin, Nt-Spire greatly enhanced FH1_D_-FH2–induced nucleation (Figure 2A, purple line) and promoted filament assembly by FH2 (Figure 2A, magenta line). Both FH1_D_-FH2 and FH1t-FH2 nucleated assembly from PA and were stimulated by Spire quantitatively identically to the original FH1-FH2 of Fmn2 (Figure S1A,B,C).

The KIND domain and the FSI peptide each abolished the synergistic effect of FH1_D_-FH2 and Nt-Spire, indicating that enhanced promotion of actin assembly results from the direct interaction between Nt-Spire and Fmn2, as observed *in vivo* (Figure 2B). The inhibition by KIND developed in a substoichiometric fashion, suggesting that only one KIND polypeptide bound per FH2 dimer greatly alters the activity of the dimer (Figure S1D). To confirm that synergistic asssembly results from the direct interaction between Spire and FH2, we tested the ability of FH1_D_-FH2ΔFSI to stimulate actin assembly in synergy with Nt-Spire. Although deletion of the FSI greatly diminished the stimulation of actin assembly, as observed with the Capu-CT construct [25], KIND did not inhibit the residual activity of FH1_D_-FH2ΔFSI and Nt-Spire failed to stimulate it (Figure S1E). The inhibition of assembly by Nt-Spire was attributed to its competitive displacement of FH1_D_-FH2ΔFSI from barbed ends. We conclude that (1) the C-terminal region of Fmn2, like Cappuccino, plays a functional role in actin assembly and (2) both the inhibition by KIND and the stimulation by Nt-Spire of the activity of FH1_D_-FH2 are mediated by the direct interaction of the C-terminal region of Fmn2 with the KIND domain of Spire.

Puzzlingly, interaction of the FH2 domain of FH1_D_-FH2 with the isolated KIND domain of Nt-Spire makes an abortive complex for nucleation, while this interaction, in the context of Nt-Spire comprising its four WH2 domains, is required for enhanced filament assembly from PA. The opposite behaviors of KIND and Nt-Spire thus reveal that the interaction of the WH2 domains of Nt-Spire with the barbed face of actin is involved in the synergy between Nt-Spire and FH1_D_-FH2. Since in the polymerization assay G-actin is 97.5% saturated by profilin, the main candidate left for WH2 binding is an F-actin subunit at the filament barbed end. The FH1 domain of FH1_D_-FH2 or FH1-FH2 is dispensable, but improves the synergy.

In the absence of profilin, FH1_D_-FH2– or FH2-nucleated filament assembly is also stimulated by Nt-Spire, however since both formin and Nt-Spire individually nucleate actin, no clear evidence distinguishes synergistic from simple additive effects (Figure S2).

We then measured the rate of assembly in the presence of profilin, FH1_D_-FH2, and increasing concentrations of Nt-Spire (Figure 2C). The assembly rate first increased with Nt-Spire up to a maximum of 5-fold. At higher Nt-Spire concentrations, the assembly rate and the amount of F-actin assembled at steady state both decreased. The increase in unassembled actin at steady state is consistent with increasing capping of the barbed ends Nt-Spire [20]. Indeed PA complex does not assemble at pointed ends; thus, profilin becomes a G-actin sequestering protein when all barbed ends are capped. The amount of PA at steady state, [PA_SS_], then is expressed as follows [30],[31]:
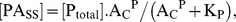
where [P_total_] represents the total concentration of profilin, A_C_^P^ the critical concentration for actin assembly at pointed ends, and K_P_ the dissociation constant of PA complex. The decreased amount of F-actin upon addition of Nt-Spire thus reflects the gradual saturation of barbed ends by Spire dominating over FH1-FH2.

The superimposed increases in the rate of assembly at a series of FH1_D_-FH2 concentrations are suggestive of a titration of FH1_D_-FH2 by Nt-Spire in an assembly-productive complex, whereas the competitive antagonism between Nt-Spire and FH1_D_-FH2 at barbed ends appears when Nt-Spire dominates over FH1_D_-FH2 (Figure 2D). A similar behavior was displayed by FH2 and Nt-Spire (Figure S3).

Spontaneous filament assembly from a large amount of monomeric actin is not a physiologically relevant process. *In vivo*, the steady state levels of assembled and unassembled actin vary via relaxation processes linked to regulatory signaling. To address the synergy between Nt-Spire, profilin, and FH1_D_-FH2 under such cellular conditions, we monitored the amount of F-actin assembled at steady state in the presence of profilin, Nt-Spire, and increasing amounts of FH1_D_-FH2. In the absence of FH1_D_-FH2, Nt-Spire caused a decrease in the amount of F-actin at steady state, due to the accumulation of PA, subsequent to barbed end capping by Nt-Spire (see above). Addition of FH1_D_-FH2 restored the amount of F-actin measured in absence of Nt-Spire (Figure 2E). Thus, FH1_D_-FH2 reversed the dominant barbed end capping effect of Nt-Spire by generating actively polymerizing barbed ends from PA. The relative amounts of unassembled and assembled actin at steady state are controlled by the Nt-Spire∶FH1_D_-FH2 molar ratio.

### FH1_D_-FH2 Associates to Nt-Spire–Capped Filament Barbed Ends to Initiate Growth from PA

**Figure 3:**
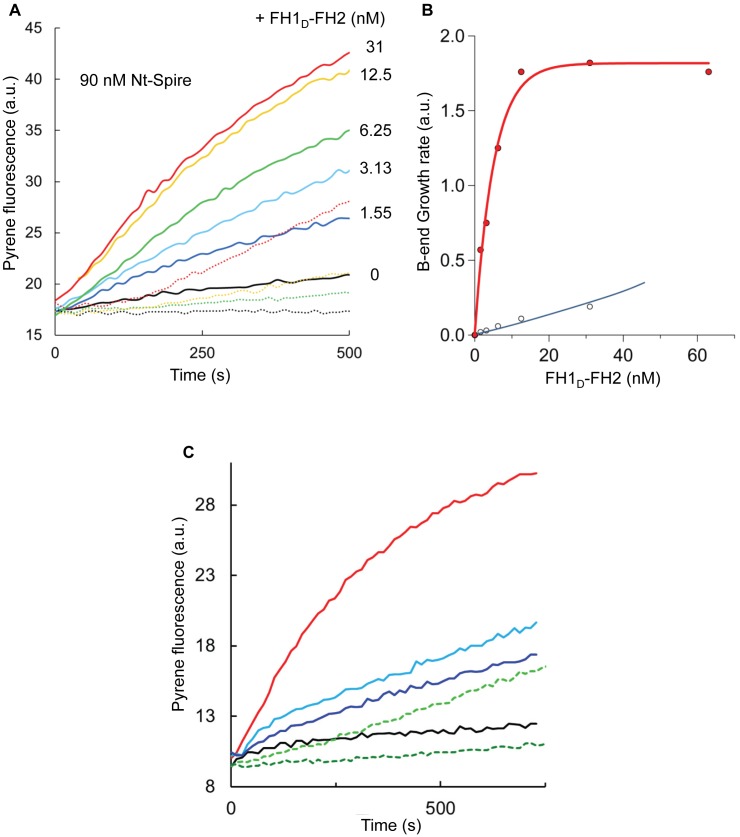
Fmn2 binds with high affinity to Nt-Spire–capped barbed ends to restore filament growth. (A) Barbed end growth from PA, initiated by 0.6 nM spectrin-actin seeds, 95% inhibited by Nt-Spire (93 nM, black line), with addition of FH1_D_-FH2 at indicated amounts in nM. Dashed lines, corresponding samples without seeds (same color coding). (B) Initial rates of barbed end growth from spectrin-actin seeds (0.6 nM; red symbols, from panel A) show a saturation behavior of Nt-Spire–bound barbed ends by FH1_D_-FH2. Open symbols, rate of spontaneous assembly under the same conditions, without seeds. (C) Seeded barbed end growth in the presence of 0.6 nM spectrin-actin seeds, Nt-Spire (93 nM) alone (black curve), and with addition of 25 nM FH1_D_-FH2 in the absence of inhibitors (red curve) and in the presence of KIND at 1.1 µM (light blue curve) or 3.5 µM (dark blue curve), in the presence of FSI at 1.1 µM (light green curve), and 2.2 µM (dark green curve).

In spontaneous assembly assays, both nucleation and barbed end growth contribute in the global polymerization rate. To understand whether only nucleation or also barbed end growth from PA is affected by FH1_D_-FH2 and Nt-Spire, seeded barbed end growth assays were performed (Figure 3A,B). Barbed end growth from PA was blocked by Nt-Spire alone (Figure 3A, black line), in agreement with previous work [20], but not detectably affected by FH1_D_-FH2 alone up to 200 nM (single filament studies described later in the text explain why). Strikingly, addition of FH1_D_-FH2 in the range 0 to 30 nM to Nt-Spire-capped filaments (90 nM Nt-Spire) restored barbed end growth to a defined level. Note that in the absence of seeds, controls show a very low level of nucleation (dotted lines in Figure 3A, blue line in Figure 3B), demonstrating that the main effect measured in the presence of seeds is on seeded barbed end growth. The FH1_D_-FH2 concentration dependence of the increase in initial rate displays a saturation behavior (Figure 3B). The very low concentration at half-effect (Kd = 2 nM) of FH1_D_-FH2 for Nt-Spire–bound barbed ends at largely saturating amounts of Nt-Spire is not consistent with the competitive displacement of Nt-Spire from barbed ends by FH1_D_-FH2. A more plausible explanation is that enhanced barbed end growth results from high affinity direct binding of FH1_D_-FH2 to barbed end-bound Nt-Spire, contrasting with its absence of effect on free barbed ends. In agreement with this interpretation, both KIND and FSI inhibited the stimulating effect of Nt-Spire on barbed end growth by FH1_D_-FH2 (Figure 3C). These bulk solution assays reveal the synergy between Nt-Spire and Fmn2 at barbed ends, but only provide an averaged measure of barbed end growth. They do not specify the number of re-growing filaments nor their individual growth rates and they do not provide information on Fmn2 processive parameters.

While ADP-actin [31],[32] and AMPPNP-actin [29] are both competent for filament assembly and profilin binding, FH1_D_-FH2 did not nucleate assembly of actin filaments from profilin-ADP-actin nor from profilin-AMPPNP-actin, and Nt-Spire did not stimulate filament assembly in either case (Figure S4). The data extend conclusions established for ADP-actin [33],[34].

### Fast Processive Assembly of Individual Filaments by Fmn2 Is Enhanced by the Transient Association of Nt-Spire and Fmn2 Together to an Individual Barbed End

Bulk solution studies demonstrate that Nt-Spire and FH1_D_-FH2 not only antagonize by competing with each other, but also bind together at barbed ends to enhance filament assembly from PA. These studies were essential in outlining the mechanistic issues and designing the appropriate conditions of assays conducted using TIRF microscopy of individual filaments, to understand how Nt-Spire and FH1_D_-FH2, individually and together, affect barbed end nucleation and assembly dynamics.

**Figure 4:**
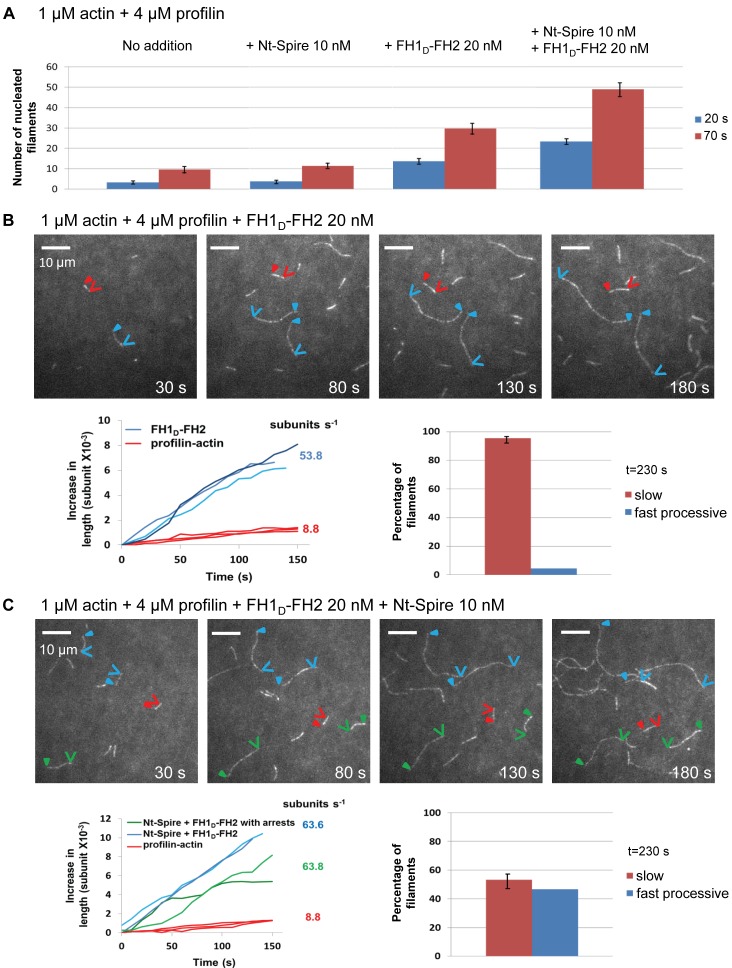
Nt-Spire interaction with Fmn2 at growing barbed ends induces rapid processive assembly events. (A) Nucleation of filaments in the presence of actin and profilin, Nt-Spire alone, FH1_D_-FH2 alone, and Nt-Spire+FH1_D_-FH2. Histograms represent the number of nucleated filaments measured by TIRF in a field of 512×512 pixels (136.7×136.7 µm) at times 20 s and 70 s after mixing all components of the sample. The bar represents standard deviation derived from triplicate assays. (B) Time lapse images of filaments elongating in the presence of 1 µM PA (10% Alexa488-labeled) and 20 nM FH1_D_-FH2. Triangles and arrows point to pointed and barbed ends, respectively, of filaments elongating slowly in the free state (red), and rapidly in the FH1_D_-FH2–bound processive state (blue). The graph represents the traces of filaments elongating slowly (red) and fast (blue). The histogram represents the percent of filaments in each class (*N* = 150–170). (C) Time lapse images of filaments elongating in the presence of 1 µM PA, FH1_D_-FH2, and Nt-Spire. When Nt-Spire was present, in the period of observation some filaments (green) displayed alternating pausing and fast elongation phases. Graph and histogram are derived as described in (B). All experiments are performed in triplicate; bars represent standard deviation.

Filament nucleation was monitored by TIRF in the presence of PA alone and with addition of Nt-Spire, or FH1_D_-FH2, or both together (Figure 4A). Nucleation was stimulated by FH1_D_-FH2 and enhanced by addition of Nt-Spire. In the presence of PA alone, filaments grew slowly (8.8±1.3 subunits per second, *N* = 20). Upon addition of FH1_D_-FH2 (20 nM), rare very fast elongation events (53.8±6.5 subunits per second, *N* = 20) over periods of up to 2 min were observed (Figure 4B, Movie S1), while 95% of filaments grew slowly at the rate characteristic of free barbed ends. Hence, by itself FH1_D_-FH2 is processive, but rarely binds to free barbed ends. In the presence of PA, 10 nM Nt-Spire and 20 nM FH1_D_-FH2, 47% of filaments displayed fast sustained growth with the same rate (63.6±6.3 subunits per second, *N* = 20) as with FH1_D_-FH2 alone (Figure 4C). Some of these filaments showed alternating periods of fast growth (63.8±11.7 subunits per second, *N* = 7) and arrested growth (green traces, Figure 4C and Movie S2). Thus, Nt-Spire facilitates FH1_D_-FH2–induced fast processive events.

**Figure 5:**
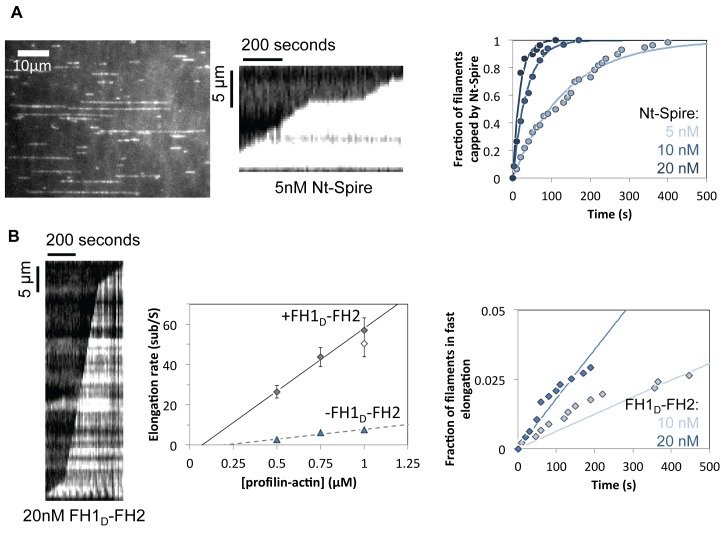
Microfluidics-assisted TIRF microscopy analysis of the binding of Nt-Spire and Fmn2 to free barbed ends. All data are with 1 µM MgATP-G-actin, 4 µM profilin. (A) Binding of Nt-Spire to free barbed ends. (Left) Typical field. (Center) Kymograph of a filament growing in the presence of 5 nM Nt-Spire. (Right) Time dependence of the fraction of filament barbed ends that are capped by Nt-Spire at the indicated concentrations. (B) Binding of FH1_D_-FH2 to free barbed ends. (Left) Kymograph of filament growing in the presence of 20 nM FH1_D_-FH2. (Center) Dependence of the rate of barbed end processive growth on PA concentration (3 µM excess profilin over a 1∶1 molar ratio to actin). Triangles, free barbed ends. Closed diamonds, FH1_D_-FH2–bound barbed ends. Open diamond, FH1-FH2–bound barbed ends. (Right) Time dependence of the fraction of filament barbed ends that start rapid processive assembly at the indicated FH1_D_-FH2 concentrations.

**Figure 6:**
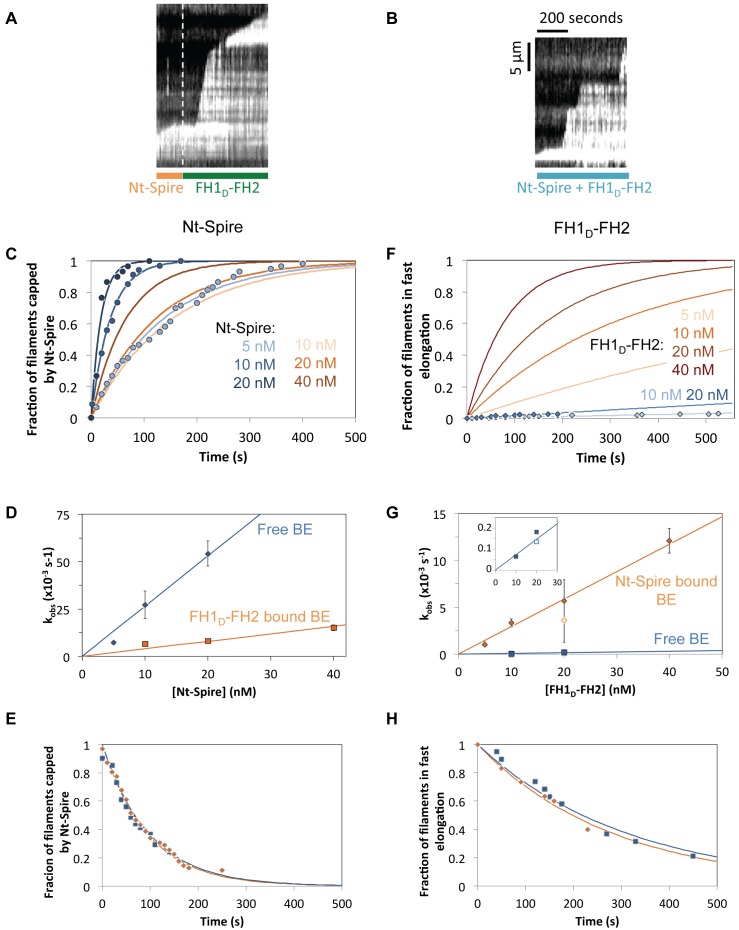
Interplay between Nt-Spire and FH1_D_-FH2 at barbed ends. Kymographs of a filament growing sequentially from PA (1 µM actin, 4 µM profilin) in the presence of (A) 10 nM Nt-Spire, then 20 nM FH1_D_-FH2, no Nt-Spire; (B) 10 nM Nt-Spire and 20 nM FH1_D_-FH2 together. (C, D, and E) Compared kinetic analysis of Nt-Spire binding to free (blue lines) and FH1_D_-FH2–bound barbed ends (orange lines). (C) Time course of the fraction of barbed ends in arrested growth. The orange curves are deduced from the direct observations of filaments switching from a rapidly growing FH1_D_-FH2–bound state to arrest, taking into account the effect of the spontaneous dissociation of FH1_D_-FH2 from the barbed end (Figure S5A). (D) Derived observed first order rate constant versus Nt-Spire concentration. (E) Fraction of filaments remaining in a regime of arested growth upon removal of Nt-Spire, following association of Nt-Spire to a free barbed end (blue symbols) or to a FH1_D_-FH2–bound barbed end (orange symbols). (F, G, H) Compared kinetic analysis of FH1_D_-FH2 binding to free (blue lines) and Nt-Spire–bound barbed ends (orange lines). (F) Fraction of filaments capped by Nt-Spire (arrested growth). The orange curves are deduced from the direct observations, taking into account the effect of the spontaneous dissociation of Nt-Spire from the barbed end (Figure S5B). (G) Derived first order rate constant versus FH1_D_-FH2 (closed symbols) or FH1-FH2 (open symbols) concentration. (Inset) Enlarged view of data for binding of FH1_D_-FH2 or FH1-FH2 to free barbed ends. (H) Fraction of filaments remaining in fast growth following removal of FH1_D_-FH2 and following FH1_D_-FH2 binding to a free barbed end (blue symbols) or to a Nt-Spire–bound barbed end (orange symbols).

The mutual interplay of the two proteins at individual barbed ends was quantified by kinetic experiments using microfluidics-assisted TIRF microscopy (Figures 5 and 6). This method allows to monitor changes in filament growth rate within 1 s delay following a change in solution conditions [34],[35].

**Figure 7:**
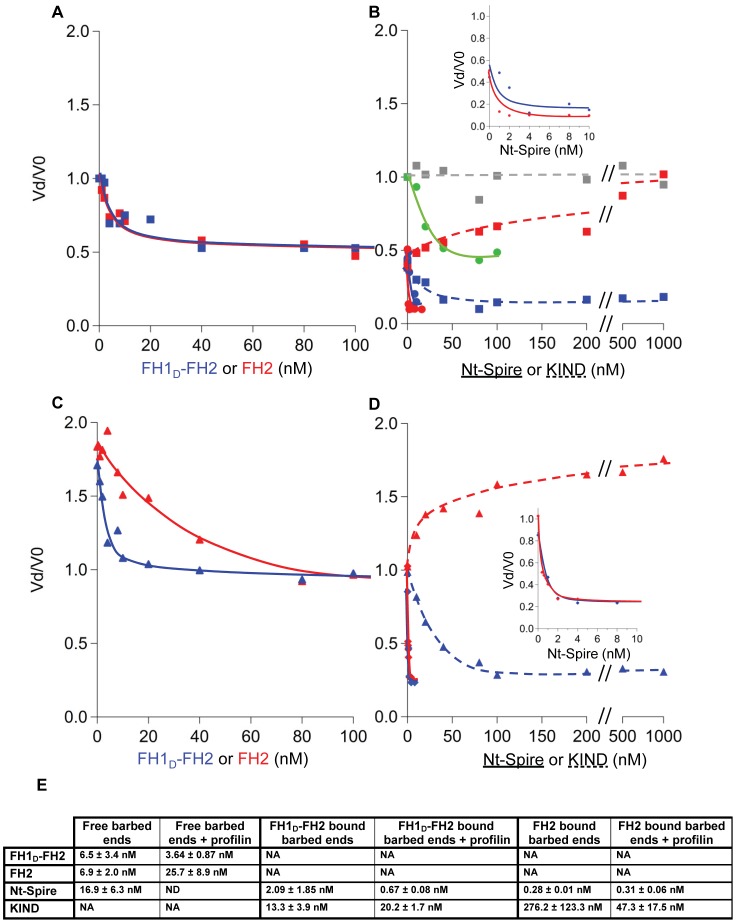
Nt-Spire and Fmn2 associate in a high affinity complex at barbed ends to block filament disassembly. (A) Initial rate of depolymerization from barbed ends in the presence of FH1_D_-FH2 (blue) or FH2 (red). (B) Initial rate of depolymerization in the presence of 100 nM FH2 (red) or FH1_D_-FH2 (blue), with addition of KIND (dashed lines) or Nt-Spire (continuous lines, and expanded scale in the inset). Control experiments show the effect of KIND (grey) and Nt-Spire (green) in absence of either FH2 or FH1_D_-FH2. (C) Initial rate of depolymerization from barbed ends in the presence of 15 µM profilin and FH2 (red) or FH1_D_-FH2 (blue). (D) Initial rate of depolymerization in the presence of 15 µM profilin, 100 nM FH2 (red), or FH1_D_-FH2 (blue) and addition of KIND (dashed lines) or Nt-Spire (continuous lines, expanded scale in the inset). All data in (A), (B), (C), and (D) are normalized to a value of 1 for the rate of free barbed end depolymerization in F buffer. (E) Table summarizing the binding parameters of Nt-Spire, KIND, FH2, and FH1_D_-FH2 to ADP-bound barbed ends in the presence and absence of profilin, derived from data shown in (A), (B), (C), and (D).

The rate of association of Nt-Spire to barbed ends was revealed by the time taken for filaments to switch from slow growth in the presence of PA to arrested growth (growth rate = 0), following addition of Nt-Spire to the flowing PA solution (Figure 5A, Movie S3). A kymograph of the capping of one filament by Spire (5 nM) is shown in central frame (Figure 5A). The apparent first order rate constant for Spire binding to barbed ends was measured at different concentrations (Figure 5A, right frame). The rate constant for Spire association to barbed ends was derived from the linear dependence of the pseudo–first order rate constant on Spire concentration. Conversely, dissociation of Nt-Spire from capped barbed ends was revealed by the switch from arrested growth to restored slow growth of free barbed ends from PA upon changing the flowing solution from PA+Nt-Spire to PA alone. Values of 2.7 µM^−1^ s^−1^ and 0.0101 s^−1^ were found for the association (k_+S_) and dissociation (k_−S_) rate constants of Nt-Spire at free barbed ends (Figure 5A) from which the equilibrium dissociation constant of Nt-Spire for barbed ends is K_S_ = k_−S_/k_+S_ = 3.8 nM. This value is in reasonable agreement with our previous bulk solution measurements demonstrating capping of barbed ends by Spire [20], further documented here, (Figure 7).

The association of FH1_D_-FH2 to free barbed ends, revealed by the switch from slow to fast growth, was addressed using the same protocol (Figure 5B). The association of FH1_D_-FH2 to free barbed ends was so slow that very few fast growing filaments were recorded over a period of 10 min, in contrast with mDia1 (our unpublished observations) and Capping Protein [36]. The measured association rate constant of FH1_D_-FH2 to free barbed ends was k_+F_ = 7.4 10^−3^ µM^−1^ s^−1^ (Figure 5B). The off rate constant of FH1_D_-FH2 derived from the duration of processive growth was k_−F_ = 3.17 10^−3^ s^−1^, consistent with an average dwell time of FH1_D_-FH2 at barbed ends of 3 to 4 min at 1 µM PA (corresponding to processive assembly of a 37 µm long filament). The rate of fast growth increased linearly with PA concentration, leading to a rate constant of 63±4 µM^−1^ s^−1^ for processive assembly by FH1_D_-FH2 from PA (Figure 5B), compared with the value of 48 µM^−1^ s^−1^ for mDia1, so far the fastest known formin [37]. Quantitatively identical data were obtained with FH1-FH2 (Fmn2), indicating that the FH2 domain of formin 2, not the FH1 domain, is responsible for its intrinsic processive behavior (open symbol in Figure 5B, central panel, inset of Figure 6G, and table in Figure S5D).

In more complex assays, filaments first capped by Nt-Spire were switched to the same solution of PA containing FH1_D_-FH2 either in absence or presence of Nt-Spire (kymographs in Figure 6A,B and Figure S5).

These assays revealed major striking features of the synergy between Nt-Spire and FH1_D_-FH2. Remarkably, each of the two proteins associated with a barbed end occupied by the other. Binding of Nt-Spire to FH1_D_-FH2–bound, rapidly growing barbed ends caused arrest of fast growth. Binding of FH1_D_-FH2 to Nt-Spire–arrested barbed ends promoted fast growth. Nt-Spire associated to a FH1_D_-FH2–bound barbed end more slowly than to a free barbed end, with a rate constant k′_+S_ = 0.396 µM^−1^ s^−1^ (Figure 6C,D, red lines; Figure S5A), as might be anticipated from the partial occupancy of barbed end subunits by structural elements of FH1_D_-FH2, hindering WH2 binding sites. In contrast, association of FH1_D_-FH2 (as well as FH1-FH2) to Nt-Spire–precapped barbed ends was 30-fold faster than to free barbed ends, leading to k′_+F_ = 0.29 µM^−1^ s^−1^, conspicuously similar to the association rate constant of Nt-Spire to FH1_D_-FH2–bound barbed ends. Ninety percent of precapped filaments displayed fast processive growth within 2 min following addition of 40 nM FH1_D_-FH2 (Figure 6F,G, red lines; Figure S5B). Identical rates of fast growth were recorded when FH1_D_-FH2 associated to a Nt-Spire–bound barbed end (57.6±6.1 subunits per second, *N* = 106) and to a free barbed end (55.5±5.9 subunits per second, *N* = 40) as in the absence of flow. Filament barbed ends were capped by Nt-Spire in the presence of FSI peptide at the same rate as without FSI (Figure S5D). However, FH1_D_-FH2 binding to barbed ends capped by Nt-Spire in the presence of FSI was strongly reduced (Figure S5C). These results establish that direct interaction between barbed end–bound Nt-Spire and Fmn2, via the KIND-FSI contact, is required to facilitate binding of Fmn2 to barbed ends and resumed fast growth. The data rule out the possibility that the synergy results only from an indirect effect of Spire binding to barbed ends. However, they do not exclude the possibility that the structure/reactivity of barbed ends is affected by the WH2 domains of Spire in a way that facilitates binding of Fmn2.

Filaments growing in the presence of both FH1_D_-FH2 and Nt-Spire displayed alternating phases of fast growth and arrested growth, visualized by staircase-like kymographs (Figure 6B). No slow growth periods were observed, suggesting that the barbed ends were never free. Arrests of growth and switches to fast growth were indicative of barbed end ocupancy by Nt-Spire and FH1_D_-FH2, respectively.

Do Nt-Spire and FH1_D_-FH2 remain bound to each other at the same barbed end, though in functionally different configurations, during the alternating periods of fast growth and arrested growth? The identical rates of FH1_D_-FH2–catalyzed processive assembly in absence or presence of Nt-Spire already argue against this possibility. We also figured that Nt-Spire (respectively FH1_D_-FH2) would dissociate from barbed ends at different rates whether it was or was not bound to FH1_D_-FH2 (respectively, Nt-Spire). Measurements of the dwell times of FH1_D_-FH2 at filaments precapped by Nt-Spire and of Nt-Spire at filaments previously in the fast growth phase before arrest unambiguously show that FH1_D_-FH2 and Nt-Spire dissociate from these preoccupied ends at the exact same rates as from free barbed ends (Figure 6E,H). Kinetic parameters are summarized in Figure S5D.

These results altogether convey the view that Nt-Spire associates directly to barbed end–bound FH1_D_-FH2, and FH1_D_-FH2 associates to barbed end–bound Nt-Spire, in transient ternary complexes. Thus, in the presence of Nt-Spire and FH1_D_-FH2, filaments switch rapidly from a pausing, Nt-Spire–capped state to a fast-growing FH1_D_-FH2–bound state, the two proteins kicking off each other to occupy their genuine binding sites at the barbed ends.

### Nt-Spire and Fmn2 Bind Tightly Together to Cap Depolymerizing ADP Barbed Ends

In filament growth assays in ATP, the nucleotide bound to barbed end subunits is ATP or ADP-Pi [32],[38]. Dilution-induced filament disassembly assays were performed to know how FH1_D_-FH2 and Nt-Spire interact with ADP-bound barbed end subunits in the absence (Figure 7A,B) and presence (Figure 7C,D) of profilin.

In the absence of profilin in the depolymerization buffer, FH2 and FH1_D_-FH2 identically slowed down filament disassembly by 50%, corresponding to about 60% inhibition of barbed end disassembly (Figure 7A). The inhibition of depolymerization occurred within 5 s mixing dead time. The formin concentration dependence of the depolymerization rate was consistent with high affinity binding of FH2 or FH1_D_-FH2 to barbed ends (K_D_ = 6±1 nM) causing a slow dissociation of ADP-actin. The rapid, high affinity binding of FH1_D_-FH2 to ADP-bound barbed ends contrasts with its slow association with growing ATP-bound barbed ends (Figure 7A). When barbed ends were saturated by FH1_D_-FH2, KIND blocked barbed end disassembly, again indicating that it bound to FH1_D_-FH2 barbed ends with a K_D_ of 20 nM and the FH1_D_-FH2-KIND complex acts as a barbed end capper (Figure 7B, dashed blue curve). Strikingly, KIND had the opposite effect on disassembly of FH2-bound barbed ends and restored the fast rate of disassembly of free barbed ends (Figure 7B, dashed red curve). Thus, binding of KIND to barbed end–bound FH2 weakens FH2 interaction with barbed end terminal subunits and promotes its dissociation from barbed ends in an inactive KIND-FH2 complex, allowing the free barbed ends to depolymerize (Figure 7B, dashed lines). KIND in itself does not affect barbed end disassembly (Figure 7B, grey curve).

The binding of Nt-Spire to barbed ends (with a K_D_ of 9 nM) slows down barbed end disassembly by about 70% (Figure 7B, green curve, and [20]). In the presence of saturating amounts of FH1_D_-FH2 or FH2 in depolymerizing buffer, which slow down disassembly by 60%, addition of Nt-Spire promoted complete blockage of barbed ends (Figure 7B, solid blue and red curves, and expanded inset). The dependence of the decrease in depolymerization rate on Nt-Spire concentration reflects the binding of Nt-Spire to FH1_D_-FH2– or FH2-bound barbed ends with 10-fold enhanced affinity (Kd = 0.5 to 1 nM) as compared to its binding to free barbed ends. Thus, Nt-Spire and FH1_D_-FH2 bind together to ADP-bound barbed ends in a configuration in which filament disassembly is blocked.

### Synergy Between Profilin, Fmn2, and Nt-Spire at Barbed Ends in a Regime of Disassembly

When profilin was present in the depolymerization buffer, FH1_D_-FH2 again slowed down filament disassembly. The dependence of the disassembly rate on FH1_D_-FH2 concentration shows that FH1_D_-FH2 binds to barbed ends with a higher affinity (Kd = 1 to 2 nM) in the presence than in the absence of profilin (Figure 7C, Figure S6A). In contrast, the affinity of the FH2 domain for barbed ends was lowered by profilin (Kd = 20 nM, Figure 7C, Figure S6B). Thus profilin strengthens the binding of FH1_D_-FH2 at barbed ends, presumably via the known interaction of profilin with the FH1 domain [39]. The effects of Nt-Spire and KIND observed in Figure 7B were conserved in the presence of profilin (Figure 7D). In conclusion, the strong interaction of FH1_D_-FH2 and Nt-Spire at ADP-bound barbed ends involves contacts between the WH2 domains of Nt-Spire and barbed end terminal subunits, in addition to the contacts between the KIND domain of Nt-Spire and the FH2 C-terminus.

Profilin enhanced the rate of disassembly from free, FH2-bound, or FH1_D_-FH2–bound barbed ends (Figure S6C), as previously observed at free barbed ends [32],[33],[40]. At saturation by profilin, slower maximal rates of depolymerization were observed in the presence than in the absence of FH2 or FH1_D_-FH2. Values of equilibrium dissociation constants of all proteins with barbed ends are summarized in the table in Figure 7E.

### Injection of Nt-Spire, FH1_D_-FH2, KIND, and FSI in Mouse Oocytes Affect Cytoplasmic Actin Asssembly Consistent with *in Vitro* Measurements

**Figure 8:**
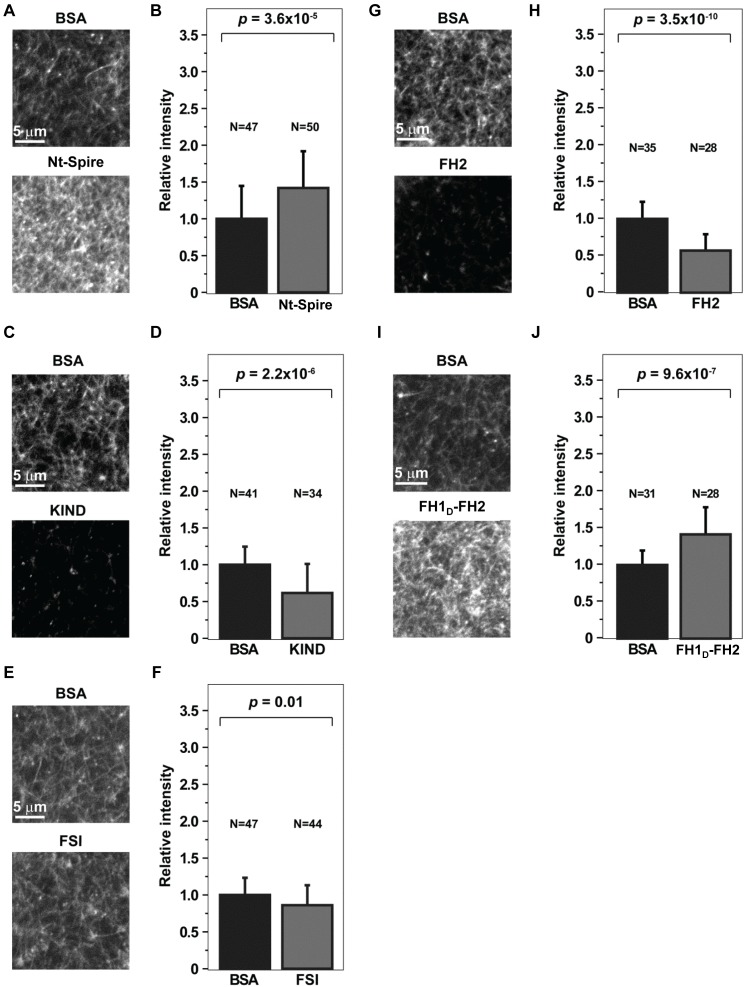
Effect of microinjection of Spire and Fmn2 protein fragments on the cytoplasmic actin network density of mouse oocytes. Confocal microscopy images of the F-actin cytoplasmic network (Alexa Fluor-488 phalloidin) in fixed oocytes microinjected with Nt-Spire (A), KIND (C), FSI (E), FH2 (G), or FH1_D_-FH2 (I). The bar graphs (B, D, F, H, and J) show the intensity of Alexa Fluor-488 phalloidin measured from a single section in the equatorial region of the oocyte. Data were pooled from three independent experiments for FH1_D_-FH2, FH2, and KIND, four independent experiments for FSI, and six independent experiments for Spire protein microinjections. Error bars indicate standard deviation.

To investigate whether the direct interaction between Nt-Spire and FH1_D_-FH2 also leads to synergistic actin assembly *in vivo*, the Nt-Spire or the isolated KIND domain, or FH1_D_-FH2, or the FSI peptide, were injected into mouse oocytes (Figure 8). Injection of Nt-Spire or FH1_D_-FH2 induced a large increase in the mass of cytoplasmic F-actin and 50% increase in intensity of fluorescent phalloidin staining as compared to the control, whereas injection of the KIND domain had the opposite effect and depressed by 2-fold the intensity of phalloidin staining indicative of cytoplasmic F-actin. Thus, constitutively active Nt-Spire and FH1_D_-FH2 recapitulate the effects of overexpression of full-length Spire and Fmn2 [6].

In the oocyte, only a fraction of the Spire and Fmn2 molecules may be bound to each other; hence, addition of constitutively active Nt-Spire or FH1_D_-FH2 may stimulate further actin assembly. In contrast, injection of KIND prevents the synergistic effect of Spire and Fmn2 on barbed end nucleation and growth. Thus, existing filaments disassemble. In agreement with our *in vitro* data showing that FH2 cannot promote processive filament assembly from PA, injection of FH2 depresses actin assembly. This result validates the concept that profilin is a player in the synergy between Spire and Fmn2.

## Discussion

### Spire and Fmn2 Regulate Processive Assembly from PA with a “Ping-Pong” Mechanism

**Figure 9:**
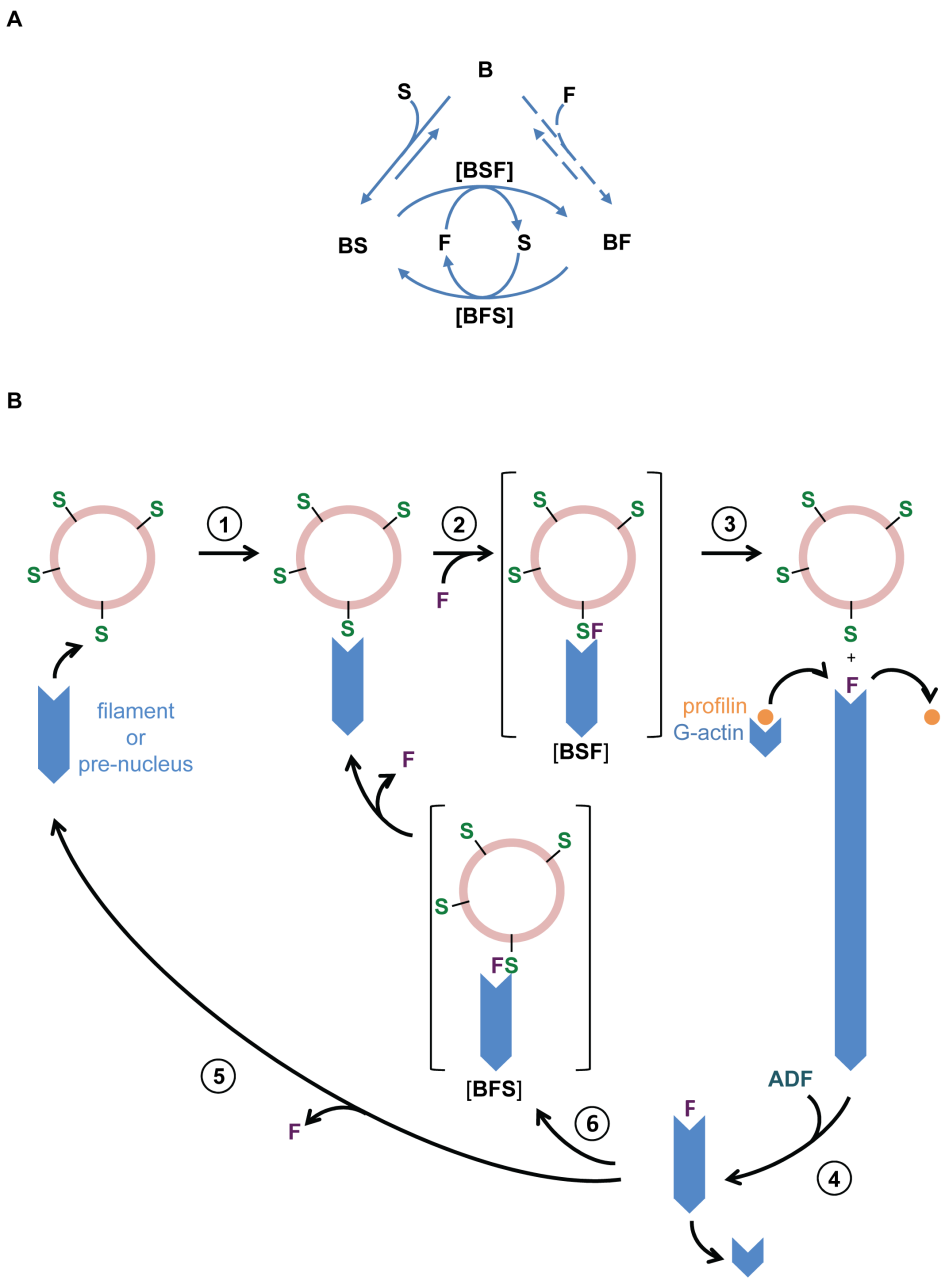
Sketch for the synergy between Nt-Spire and Fmn2 in processive barbed end filament assembly and blockage of disassembly. (A) The ping-pong mechanism. A filament barbed end (B) in the presence of Spire (S) and Fmn2 (F) associates faster to Nt-Spire than to Fmn2, leading to BS state. BS interacts much faster than B with Fmn2. Displacement of S leads to processive fast growth from BF. Binding of Spire to BF leads to dissociation of Fmn2 and establishment of the capped, nongrowing BS state. Filaments transit between the BS and BF states at frequencies governed by the amounts of Spire and Fmn2. (B) Model for organisation of a dynamic nonpolarized actin meshwork from Spire-bound vesicles in the presence of Fmn2. The following reactions are drawn. (1) attachment of a filament or nucleus barbed end to a Spire-vesicle; (2) Fmn2 is recruited by Spire at vesicle-attached barbed ends (BSF transient state); (3) Fmn2-catalyzed fast processive growth of the filament from PA; (4) ADF-promoted shortening of filaments enhances treadmilling; (5) dissociation of Fmn2 leads to recycling of barbed ends to Spire-vesicle; and (6) recycling of Fmn2-bound barbed ends to a Spire-vesicle (BFS transient state). Myosin Vb-driven translocation of Rab11a vesicles along Fmn2-assembled filaments implicitly contributes in coordinating the vesicle-filaments network dynamics, however Rab11a-activated myosin Vb is not represented for simplicity.

Bulk solution studies and single filament analysis of actin assembly provide mechanistic insight into the reported genetic interactions between Spire, Fmn2/Cappuccino, and profilin in oogenesis. The data reveal how Nt-Spire and FH1_D_-FH2 both cooperate and antagonize in filament assembly from PA, and establish that replacing the FH1 of Fmn2 by FH1_D_ of mDia1 or deleting a few proline regions does not affect the function of Fmn2 nor its synergy with Nt-Spire. Thus, the conclusions of this work apply to FH1-FH2 (Fmn2). FH1-FH2 is highly processive in itself, but binds filament barbed ends inefficiently. Capping of barbed ends by Nt-Spire kinetically facilitates barbed end association of FH1-FH2. All data emphasize that the faster binding of FH1-FH2 is due to the direct interaction between the two proteins at barbed ends rather than to only an indirect effect of the WH2 domains of Nt-Spire on the conformation of the barbed end (Figure 9A). Spire and FH1-FH2 control filament assembly using a “ping-pong” [41] (or “tag-team”) mechanism that has no precedent in the regulation of formin-mediated actin assembly. Filaments display alternate phases of fast processive growth and arrested growth, in which barbed ends bind in turn FH1-FH2 or Nt-Spire, respectively. Each protein kicks off the other via formation of transient complexes in which they interact together at the barbed end. The dwell time in each phase, as well as the relative amounts of F-actin and G-actin at steady state, are governed by the relative concentrations of Nt-Spire and FH1-FH2. The control of actin assembly dynamics by the Nt-Spire∶FH1-FH2 ratio may extend to the synergy between Nt-Spire and Cappuccino in *Drosophila* mid-oogenesis.

The following minimal scheme describes the data without making any mechanistic hypotheses.
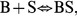
(1)

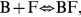
(2)

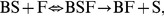
(3)

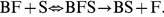
(4)B, BS, and BF represent the barbed ends in free, Nt-Spire–bound, and FH1-FH2–bound states, respectively. BFS and BSF are transient states in which Nt-Spire and FH1-FH2 interact directly together as well as with terminal subunits at the barbed end.

When Nt-Spire and FH1-FH2 coexist with PA in solution, because association of FH1-FH2 to barbed ends or prenuclei is extremely slow, a likely sequence of events (Figure 9A) is the initial rapid capping of barbed ends or prenuclei by Nt-Spire, followed by rapid association of FH1-FH2 in a low affinity transient complex BSF, leading to dissociation of Nt-Spire and formation of BF. In other words, FH1-FH2 is firmly saddled on a barbed end nucleus or filament by Nt-Spire. Spire thus assists Fmn2, in agreement with genetic data [15]. Note that the origin of the synergistic action of Nt-Spire and FH1-FH2 derived from the present data contrasts with the anticipated mechanism within the alternate view that both Spire and Fmn2 are nucleators individually, and that their interaction leads to inhibition of actin assembly [23],[25],[42].

The mutual kick off of Nt-Spire and FH1-FH2 from barbed ends implies that the transient complexes BSF and BFS differ structurally/chemically, so as to lead to BF and BS, respectively. Thus, the present data, illustrated by this scheme, raise structural and mechanistic issues regarding the possible conformations of the FH2 domain of Fmn2 and the WH2 domains of Spire interacting with the terminal barbed ends subunits of the actin filament, individually and together.

A “kick off” process may imply that each protein interacts with the barbed end with at least two subsites, which in the present case may be facilitated by the fact that two actin subunits are exposed at the filament barbed end. For instance, uncapping of capping protein (CP) from barbed ends by VopF is possible because the ß-tentacle of CP occupies the main WH2 binding site only on the terminal barbed end subunit, leaving the homologous site on the subterminal subunit available for one WH2 domain of the dimeric VopF [43]. Similarly, the crystal structure of the FH2 domain of Bni1 in complex with TMR-actin shows that the “knob” of FH2 occupies the WH2 binding site only on the subterminal subunit, leaving the barbed face of the terminal subunit exposed in the “closed” state [44]–[46]. Assuming that a large fraction of the FH2 of Fmn2 shares the actin binding mode of Bni1, it is tempting to suggest that one WH2 domain of Nt-Spire binds to the terminal subunit in the “closed” FH2-actin state, following association of the KIND domain with the C-terminal region of FH1-FH2. We find that the isolated KIND domain causes destabilization of FH2 from the barbed end, which implies that the C-terminus of FH2, which is specific to Fmn2, participates in the interaction of the FH2 domain with terminal subunits and processive walk, in agreement with Vizcarra et al. [25]. Therefore, when Nt-Spire binds to an FH1-FH2–bound barbed end, the structural change linked to FH2-KIND interaction may be involved in the kick off of FH1-FH2 coupled to tightening of Nt-Spire binding to terminal subunits. The proposed rapid equilibrium of FH2 between the “closed” and “open” states during processive assembly may be affected by Spire and may allow FH1-FH2 and Nt-Spire to adopt different conformations in BFS and BSF states as well.

The nature of the nucleotide bound to the two actin barbed end subunits may be important in the binding of FH1-FH2 and the kick off mechanism. The fact that FH1-FH2 associates very slowly to barbed ends in a regime of growth in ATP, while it binds rapidly and with high affinity to ADP-bound barbed ends, may suggest that FH1-FH2 has a higher affinity for ADP-actin, which is not frequently present at barbed ends growing from profilin-ATP-actin. Alternatively, FH1-FH2 association to ATP-bound barbed ends may occur as a two step reaction, formation of a rapid equilibrium low affinity complex being followed by a structural change strengthening the binding of FH1-FH2 and allowing processive assembly. The observation that no stimulation of filament assembly by either FH1-FH2 or FH1-FH2+Nt-Spire takes place in AMPPNP nor ADP further suggests that ATP hydrolysis plays some role in Fmn2 function as well as in its cooperation with Spire. While evidence has been provided for processive tracking of barbed ends by formins mDia1 and Bni1 in a growth regime in ADP and in a depolymerization regime [36],[46],[47], thus demonstrating that ATP hydrolysis is not required for tracking of barbed ends by formin, the very fast processive assembly from PA is oberved only in ATP [29],[33], and pauses in growth are observed upon addition of CrATP that does not release Pi following cleavage of ATP [32]. Moreover, processive assembly can be modeled without involving ATP hydrolysis only if the affinity of profilin for ATP-actin is assumed to be 50-fold lower than its acknowledged value [48].

Two other formins, INF2 and FMNL3, use WH2 domains and FH2 domains in the same polypeptide chain, to regulate actin assembly. Remarkably, in this case, the WH2 domain affects nucleation using a different mechanism [49],[50], in which interaction of the WH2 domain with G-actin relieves the auto-inhibition [51].

### Relevance of the Biochemical Interplay Between Nt-Spire and Fmn2 in Asymmetric Division

Most formins promote processive filament assembly in a Rho GTPase-mediated, site-directed fashion. In the mouse oocyte, Fmn2, which is not regulated by Rho GTPases, is recruited via Spire to Rab11a positive vesicles. Both the high dynamics of filament assembly and the action of myosin Vb, linked to Rab11a vesicles, are required for spindle translocation [12],[52]. Myosin Vb, together with Nt-Spire and Fmn2, controls the global dynamics of this coupled vesicle-filament system leading to outward movement of vesicles and slow spindle translocation toward the cortex [52].

We tentatively propose that the ping-pong mechanism integrates this context as follows (Figure 9B). Association of Nt-Spire to Rab11a vesicles leads to barbed end binding of filaments or prenuclei, triggering Fmn2 association to the Nt-Spire–attached barbed ends, displacement of Nt-Spire from the transient BSF state, and fast barbed end growth. The presumed presence of ADF/cofilin ensures rapid pointed end disassembly of the filaments, which creates a stationary large pool of PA, which feeds fast barbed end processive assembly and fosters rapid treadmilling at the scale of individual filaments [29]. The shortened filaments then either release Fmn2 spontaneously and again get capped by Nt-Spire at the surface of the vesicles, or directly bind Nt-Spire vesicles into the BFS state, then release Fmn2. For simplicity, the cycle of filament nucleation release at Rab11a vesicles organized by Spire and Fmn2 is illustrated at the level of an individual filament in Figure 9B. At the collective level, dynamic links between the filaments are imposed in part by the ping-pong mechanism and in part by the clustering of the players Nt-Spire, Fmn2, and myosin Vb at Rab11a-positive vesicles. These connections organize the formation and maintenance of a dynamic gel in a rapid renewal state that controls the plasticity of the oocyte cytoplasm and facilitates break of symmetry and the first slow step in directional migration of the spindle [53]. This process appears hampered in a gel in which filaments do not undergo rapid turnover, as demonstrated by the failure of spindle to translocate in jasplakinolide-treated oocytes [12]. The very slow migration rate of the spindle toward the cortex actually argues for a mechanism in which actin assembly in the oocyte is not directly applied to a surface to develop a pushing force. Microrheological studies of actin solutions in the presence of Nt-Spire, FH1-FH2, profilin, and ADF, mimicking cellular media, may reveal how the Nt-Spire∶FH1-FH2 balance affects the properties of this gel. A confined environment may further affect rheological properties [54].

In *Drosophila* oocytes, massive actin assembly at midoogenesis, resulting from the synergy between formin Cappuccino and Spire, is required to avoid premature cytoplasmic streaming and failure in axis patterning. The rescue of Spir mutants by expression of SpirD [15], which is identical to the Nt-Spire protein studied here, further establishes the *in vivo* relevance of the present biochemical data. Completion of oogenesis requires the subsequent disappearance of the actin meshwork. Our work shows that an excess of Nt-Spire over FH1-FH2 causes capping of barbed ends by Nt-Spire that leads to depolymerization of F-actin by profilin. Monitoring the evolution of the Spire∶Fmn2 ratio during oogenesis and manipulating it genetically may validate or rule out this potential regulatory mechanism.

## Materials and Methods

### Plasmid Constructs

The following constructs of human Spire 1 (accession number NP_001122098), mouse Formin 2 (accession number NP_062318.2), and mDia1 (accession number NP_031884) were designed as follows. FH1_D_-FH2 (P854-T1578) and truncated FH1t-FH2 (P854-T1578Δ(912–967)) constructs, FH2 (F1128-T1578) and KIND (G35-S257) cDNA were cloned between BamH1 and Xho1 cloning sites of a modified pGEX-6P1 expression vector containing a N-terminal histidine thioredoxine tag in place of the GST tag and a C-terminal Streptag II. The cDNA of the chimeric FH1(mDia1)-FH2(Fmn2), called FH1_D_-FH2, was chemically synthetized from the amino acid sequence obtained by juxtaposing the FH1 amino acid sequence of mDia1 (S568-P747) to the FH2 amino acid sequence of Fmn2 (F1128-T1578) and back-translating it to a nucleotide sequence optimized for expression in *E. coli*. The FH1_D_-FH2ΔFSI construct was subcloned from the FH1_D_-FH2 cDNA sequence down to S1558 (thus deleting the last 20 residues of the FH2 domain) into the modified pGEX-6P1 expression vector. The Nt-Spire cDNA sequence corresponding to (M1-S443) was cloned in an unmodified pGEX-6P1 vector between the BamH1 and Xho1 cloning sites.

### Expression and Purification of Fmn2 Constructs

All constructs were expressed in *E. coli* Rosetta (DE3) (Novagen), in LB medium. Cultures were induced by 1 mM IPTG at 16°C overnight. Bacteria pellet were resuspended in lysis buffer (20 mM potassium phosphate buffer pH 7.4, 900 mM NaCl, 15 mM imidazole, 3 mM DTT, 5% sucrose, 0.1 mM EDTA, 1 mM PMSF, 5 µM benzamidine, protease inhibitor cocktail, 1% Triton X100, and lyzozyme) and sonicated on ice. Ultracentrifuged cell lysates were loaded on HisTrap FF crude column (GE Healthcare). The HisTrap resin was equilibrated with binding buffer 1 (20 mM phosphate buffer pH 7.4, 900 mM NaCl, 15 mM imidazole, 3 mM DTT, 5% sucrose, 0.1 mM EDTA), then washed with 4% of elution buffer 1 (binding buffer 1 except for 250 mM imidazole). Proteins were eluted with a 60% elution buffer gradient step. The Fmn2-enriched fraction was then diluted with a suitable volume of 100 mM Tris pH 7.5 to decrease NaCl concentration to 300 mM and loaded to a Strep Trap HP (GE Healthcare). The resin was then washed with binding buffer 2 (100 mM Tris pH 7.5, 300 mM NaCl, 1 mM EDTA, 3 mM DTT, 5% sucrose), and bound proteins were eluted with elution buffer 2 (binding buffer 2 supplemented with 4 mM desthiobiotin). Eluted fractions were pooled and concentrated with a Vivaspin (10 kDa cutoff) and injected on a Superdex 200 16/60 (GE Healthcare) pre-equilibrated with 100 mM Tris pH 7.5, 300 mM NaCl, 3 mM DTT, 5% sucrose. Fractions corresponding to pure Fmn2 constructs were pooled, concentrated, flash frozen in liquid nitrogen, and stored at −80°C.

The very low level of expression and poor solubility of Fmn2 FH1-FH2 precluded extensive biochemical characterization. Truncation of two proline-rich regions of the FH1 domain or its replacement by the FH1 domain of mDia1 yielded over one order of magnitude higher level of expression of soluble constructs, respectively called FH1_t_-FH2 and FH1_D_-FH2. The Stokes radii of FH2, FH1_D_-FH2 and FH1_D_-FH2ΔFSI derived from gel filtration revealed their dimeric structure. Concentrations of FH2 and FH1_D_-FH2 are expressed in molarity of the protomer.

### Purification of FH2 and KIND

FH2 and KIND were expressed and purified similarly to FH1_D_-FH2 constructs up to the HisTrap purification step. Prior to the Strep Trap purification step, the histidine thioredoxine tag was cleaved using Prescission Protease (5 U/mg fusion protein) overnight at 4°C. Digested protein was then loaded to a Strep Trap HP (GE Healthcare). The resin was then washed with binding buffer 2, and bound proteins were eluted with elution buffer 2. Eluted fractions were pooled, concentrated, and loaded on a Superdex 200 16/60 (GE Healthcare) pre-equilibrated with 20 mM Tris pH 7.5, 75 mM KCl, 1 mM DTT for FH2, or 20 mM Tris pH 7.5, 100 mM KCl, 1 mM DTT for KIND. Fractions corresponding to pure FH2 or KIND were pooled and concentrated. FH2 was stored at 4°C. KIND was flash frozen in liquid nitrogen and stored at −80°C.

### Purification of Nt-Spire

Nt-Spire was expressed and purified similarly to FH1_D_-FH2 constructs up to the HisTrap purification step. The concentrated His Trap eluted material was loaded onto a desalting Hiprep 10–26 column pre-equilibrated with a desalting buffer (50 mM Tris pH 7.5, 400 mM NaCl, 1 mM DTT, 1 mM EDTA). The GST tag was cleaved by overnight incubation at 4°C of the concentrated fusion protein solution with Prescission Protease (5 U/mg fusion protein). Nt-Spire was eventually purified by gel filtration in 15 mM Tris pH 7.5, 250 mM KCl, 1 mM DTT, 1% sucrose buffer, and was kept frozen at −80°C.

### FSI Peptide

The FSI peptide comprising the 27 C-terminal residues of human Fmn2 (NP_064450.3) (E1549-T1578) was chemically synthetized (Proteogenix). We dissolved 10 mg of peptide in 500 µL Tris 20 mM, KCl 100 mM, and DTT 1 mM, and loaded it on a pre-equilibrated PD-10 desalting column. The eluted peptide fractions were stored frozen at −80°C.

### Actin Polymerization/Depolymerization Assays

Actin was purified from rabbit muscle and isolated in monomeric form in G-buffer (5 mM Tris-Cl^−^, pH 7.8, 0.1 mM CaCl_2_, 0.2 mM ATP, 1 mM DTT, 0.01% NaN_3_). Profilin and spectrin-actin seeds were purified as described [31]. Spectrin-actin seeds (0.1 µM), equilibrated in 0.3 mM NaPO4 pH 7.6, were reacted with 20 µM sulfoNHS-LC-LC-Biotin (Pierce) for 2 h at room temperature, then dialysed against 0.3 mM NaPO4 pH 7.6, 1 mM DTT buffer. Biotinylated spectrin-actin seeds were supplemented with 50% ethylene glycol and stored at −20°C.

ADP-actin was prepared by treatment of ATP-G-actin with hexokinase and glucose [31]. Briefly, Ca-ATP-G-actin (10 µM) in G buffer was supplemented with 20 µM MgCl_2_, 0.2 mM EGTA, 1 mM glucose, 10 µM Ap5A as an inhibitor of myokinase, and 15 U/ml hexokinase (Sigma). Polymerization assays were performed in the presence of ADP and Ap5A. AMPPNP-actin was prepared from ADP-actin as above, followed by addition of 1 mM AMPPNP and gel filtration on Sephadex G25 (PD10 columns, GE Healthcare) equilibrated in G_X_ buffer (G buffer containing 1 mM AMPPNP instead of ATP, 10 µM MgCl2, 1 mM glucose, and 5 U/ml hexokinase to ensure the absence of contaminating ATP in the commercial AMMPNP [55]). It was checked that 100% of G-actin was AMPPNP-bound by equilibrating the initial ATP-G-actin solution with [^3^H]-ATP (NEN), and measuring the absence of [^3^H]-ADP in the fractions of AMPPNP-G-actin eluted from the PD-10 column in G_X_ buffer. Solutions of ADP-G-actin and AMPPNP-G-actin were kept on ice and used within 6 h.

Actin polymerization/depolymerization kinetic experiments were based on fluorescence change of pyrenyl-labeled G- or F-actin (λexc = 366 nm, λem = 407 nm). All experiments were carried out at 20°C, on a Safas Xenius FLX spectrofluorimeter (Safas, Monaco), using a multiple sampler device.

Polymerization assays were performed in F-buffer (5 mM Tris-Cl pH 7.8, 0.2 mM ATP, 0.1 mM CaCl_2_, 1 mM DTT, 0.05 M KCl, 1 mM MgCl_2_). Prior to each experiment, a stock solution of CaATP-G-actin (10 µM, 5% pyrenyl-labeled) was converted into MgATP-G-actin by addition of 20 µM MgCl_2_ and 0.2 mM EGTA and kept on ice. We added 24 µM Profilin to this stock solution for polymerization assays at final concentrations of 2.5 µM G-actin and 6 µM profilin.

Dilution-induced depolymerization assays were performed by quickly diluting 4 µL of 2.5 µM 50% pyrenyl-labeled F-actin into 196 µL F buffer containing the proteins of interest. The initial rate of depolymerization was measured and normalized with respect to the initial depolymerization rate in control samples.

Measurements of F-actin asembled at steady state were performed as described [29] using 2% pyrenyl-labeled actin. Samples were incubated at 4°C overnight in the dark before fluorescence measurements.

### TIRF Measurements of Single Filaments

Standard TIRF assays were performed using a flow chamber assembled by placing two parallel strips of double-sided tape (26×10×0.1 mm) spaced by 8 mm onto a cleaned glass slide (76×26 mm), surmounted with a PLL-PEG passivated coverslip. Chambers were sequentially washed with G buffer, 5% BSA, Fluo F buffer (5 mM Tris-Cl− pH 7.8, 150 mM NaCl, 1 mM MgCl_2_, 0.2 mM EGTA, 0.2 mM ATP, 10 mM DTT, 1 mM DABCO, 0.01% NaN_3_). Assays were performed in Fluo F buffer supplemented with 0.3% methylcellulose (Sigma Cat. No. M-0262, 400 cP for a 2% aqueous solution at 20°C) and with actin, profilin, Nt-Spire, and FH1_D_-FH2 or FH1-FH2 at indicated concentrations.

Microfluidics-assisted TIRF microscopy assays were performed using PDMS flow cells, with three inlets [34]. Prior to flowcell assembly, coverslips are first extensively cleaned by sequential sonication in pure water, ethanol, and 1 M KOH for 20 min each, then dried with air and exposed to a plasma discharge for 2 min. The microchambers were placed on the microscope stage and connected to the microfluidic system (MFCS and Flowell, from Fluigent). The coverslip is then functionalyzed by absorption of PLL-PEG/PLL-PEG-biotin (20%) (from SuSoS) to minimize nonspecific protein binding and achieve specific anchoring of biotinylated spectrin-actin seeds via a streptavidin sandwich. Actin was labeled with Alexa488 succimidyl ester [34]. The fraction of labeled actin was 10%. Assays were performed in FluoF buffer without methylcellulose.

TIRF observations were carried out on an Olympus IX71 inverted microscope, with a 60× TIRF objective, and a 473 nm laser (Cobolt). The experiment was controlled using the Metamorph software. Images were acquired using a cascade II EMCCD camera (Photometrics), with a frame interval of 10 s for all experiments. Images are further analyzed by ImageJ to obtain kymographs and to determine the times at which filaments experience transitions from one to another of the three possible states: slow elongation (“free barbed-end”), rapid elongation (“FH1_D_-FH2–bound barbed end”), or capped (“Nt-Spire–bound barbed end”). Single exponential curve fitting of the data points is done using Gnuplot. On the kymographs, slopes of elongation phases give us the elongation rates in presence or absence of FH1_D_-FH2. We considered that each actin subunit contributes to 2.7 nm of the filament length.

### Preparation and Microinjection of Mouse Oocytes

All mice were maintained in a specific pathogen-free environment according to UK Home Office regulations. Oocytes were isolated from ovaries of 8-wk-old FVB mice, cultured, and microinjected as described in detail [53]. BSA (Sigma) or recombinant Nt-Spire, KIND, FH2, and FH1_D_-FH2 protein fragments were microinjected into oocytes in buffer supplemented with 0.05% NP-40 Alternative (Calbiochem). Final protein concentrations were calculated by dividing the total amount of injected protein by the total volume of the oocyte. These were 1 to 3 µM for each protein, 8 µM for KIND, and 163 µM for FSI.

### Measurement of Cytoplasmic Actin Network Density

At 4–5 h after resumption of meiosis using previously detailed methods [46], oocytes were fixed for 30 min at 37°C with 100 mM HEPES, 50 mM EGTA, 10 mM MgSO4, 2% formaldehyde, and 0.2% Triton X-100 and extracted in PBS supplemented with 0.1% Triton X-100 at 4°C overnight. Actin staining was performed for 1 h in PBS, 3% BSA, and 0.1% Triton X-100 with Alexa Fluor-488 Phalloidin (Molecular Probes; 1∶20).

Single optical sections in the equatorial region of oocytes were acquired with a Zeiss LSM710 confocal microscope equipped with a ×63 C-Apochromat 1.2 NA water-immersion objective as described previously [56]. Images in control and perturbed situations were acquired with identical imaging conditions. Care was taken that images were not saturated during acquisition. To quantify the density of the cytoplasmic actin network, the mean intensity of Alexa Fluor-488 phalloidin was measured in the cytoplasm and in a region outside the oocyte for background subtraction using ImageJ. Average (mean), standard deviation, and statistical significance based on Student's *t* test (always two-tailed) were calculated in OriginPro (OriginLab).

## Supporting Information

Figure S1 **Validation of the FH1_t_-FH2 and FH1_D_-FH2 as substitutes of Fmn2 FH1-FH2.** (A) FH1_D_-FH2 (blue lines) and original Fmn2 FH1-FH2 (green lines) display identical stimulation of actin assembly at a series of construct concentrations. (Inset) Histogram of the global asssembly rates, using same color coding as the raw data curves. (B) FH1_D_-FH2 (blue lines) and original Fmn2 FH1-FH2 (green lines) display identical synergy with Nt-Spire at a series of equimolar Nt-Spire∶FH1_D_-FH2 or Nt-Spire∶FH1-FH2 concentrations. (Inset) Histogram of the global asssembly rates, using same color coding as the raw data curves. (C) FH1_t_-FH2 (magenta lines) and original Fmn2 FH1-FH2 (green lines) display identical stimulation of actin assembly and functional interaction with Nt-Spire. (D) Dose dependence of the inhibition of FH1_D_-FH2 by KIND. (Inset) Maximal inhibition by KIND was reached at substoichiometric amount of KIND at 25 nM (light blue) and 50 nM Fmn2 (dark blue). (E) FH1_D_-FH2ΔFSI stimulates poorly filament assembly from profilin actin, is not affected by KIND, and is slightly inhibited by Nt-Spire. (Inset) The *y*-axis magnification of the actin assembly by FH1_D_-FH2ΔFSI, FH1_D_-FH2ΔFSI+KIND, and FH1_D_-FH2ΔFSI+Nt-Spire. All experiments in (A), (B), (C), (D), and (E) are with 2 µM actin, 4 µM profilin. (F) Coomassie Blue–stained SDS-PAGE of the various constructs used in the work. We loaded 30 pmoles of each protein in each lane.
(TIF)

Figure S2 **Stimulation of actin assembly by FH1_D_-FH2, Nt-Spire, and both proteins together in the absence of profilin.** Conditions are 2.5 µM actin with or without 50 nM Nt-Spire or FH1_D_-FH2 or both proteins together.
(TIF)

Figure S3 **Spire induces filament assembly from PA in the presence of FH2.** Actin (2.5 µM) was polymerized in the presence of 6 µM profilin (black line), 50 nM Spire (dotted purple line), and 50 nM FH2 without (red dotted line) or with increasing amounts of Spire (in nM).
(TIF)

Figure S4 **FH1_D_-FH2 fails to stimulate filament assembly from PA in the presence of ADP or AMPPNP.** Actin was prepared in ADP- (A) or AMPPNP- (B) bound form (Materials and Methods) and assembled in the presence of profilin without (black lines) and with 200 nM FH1_D_-FH2, in absence (blue lines) or presence of Nt-Spire (red lines). ATP was then added (arrow).
(TIF)

Figure S5 **Measurement of the association rates of Spire (resp. Fmn2) to Fmn2- (resp. Spire-) bound barbed ends.** (A) Fmn2-bound filament barbed ends (BF) are exposed to a flow of PA+Spire (without Fmn2). Arrest of rapid growth of Fmn2-bound barbed ends by Spire results from the combination of Fmn2 dissociation followed by association of Spire to a free barbed end and direct association of Spire to Fmn2-bound barbed ends, possibly followed by dissociation of Fmn2. The free barbed ends produced by the very slow reaction BF→B (rate constant k_−F_) grow slowly or are capped (transition from BF to BS). The reaction B→BS is rapid, and thus, the transition from state BF to BS includes the route BF→B→BS in addition to reaction BF→BS (with apparent rate constant k′^app^_+S_ for a given concentration of Spire). Based on the resolution of our experiment and on the rate constants k_+S_ and k_−S_, most filaments reaching state B are capped and convert to state BS very rapidly, while the vast majority (more than 90%) of the capped states BS last long enough to be identified unambiguously. Consistently, as shown at 40 nM Nt-Spire (graph), we observe almost only transitions from state BF to BS. The observed rate constant can be written k_obs_ = k_+S_[S](k′^app^_+S_+k_−F_)/(k_+S_[S]+k_−F_). The data are fitted with k′^app^_+S_ as a free parameter. The resulting k′^app^_+S_ varies linearly with [Spire] (Figure 6D). (B) Spire-capped filaments (BS) are exposed to a flow of PA+Fmn2 (without Spire). Resumed fast growth at Spire precapped barbed ends results from two combined kinetic routes, dissociation of Spire followed by association of Fmn2 to a free barbed end, and direct association of Fmn2 to a Spire-capped barbed end possibly followed by rapid dissociation of Spire. Free barbed ends enter a state of slow growth (reaction BS→B, with rate constant k_−s_). Filaments that undergo reaction BS→BF, with the apparent rate constant k′^app^_+F_ (for a given concentration of Fmn2), grow fast. The reactions B→BF and BF→B are slow, and states B and BF last long enough to be identified unambiguously in our experiment. Overall loss of capping occurs at an observed rate k_−s_+k′^app^_+F_, with a fraction k_−s_/(k_−s_+k′^app^_+F_) of the filaments undergoing reaction BS→B, and a fraction k′^app^_+F_/(k_−s_+k′^app^_+F_) of the filaments undergoing reaction BS→BF. The graph shows the measured transitions occurring over time for each class and the sum of the two, for [FH1_D_-FH2] = 10 nM (full symbols, solid lines) and 20 nM (open symbols, dashed lines), fitted using k′^app^_+F_ as a free parameter. The resulting k′^app^_+F_ is found to vary linearly with [Fmn2] (as reported in Figure 6G). (C) Filaments capped by Nt-Spire in the presence of FSI fail to associate with FH1_D_-FH2 (10 nM) and resume fast growth. The values of k_obs_ are measured as under Figure 6G. (D) Table summarizing the rate constants for FH1_D_-FH2, FH1-FH2 (italics), and Nt-Spire association and dissociation at free or FH1_D_-FH2- (resp. Nt-Spire-) bound barbed ends, and the asssociation rate constant k+ of PA to FH1_D_-FH2–bound barbed ends in fast processive assembly. The dissociation of Nt-Spire from barbed ends is not affected by FSI.
(TIF)

Figure S6 **Profilin strengthens the binding of FH1_D_-FH2 at depolymerizing barbed ends.** (A) Barbed end disassembly at the indicated concentrations of profilin is slowed down by FH1_D_-FH2 binding to barbed ends. The affinity of FH1_D_-FH2 for barbed ends is increased by profilin. (B) Barbed end disassembly at the indicated concentrations of profilin is slowed down by FH2. The affinity of FH2 for barbed ends is lowered by profilin. (C) Double reciprocal plots of the profilin concentration dependence of the rate of depolymerization from barbed ends in the absence (grey) and presence of either 100 nM FH2 (red) or FH1_D_-FH2 (blue).
(TIF)

Movie S1 **Rare fast elongation events with FH1_D_-FH2 alone (corresponding to** **Figure 4B** **, top panel).** In the presence of 20 nM of FH1_D_-FH2, rare very fast elongation events were observed. 1 frame/10 s, 20-fold acceleration.
(AVI)

Movie S2 **Spire facilitates FH1_D_-FH2–induced fast processive events (corresponding to** **Figure 4C** **, top panel).** In the presence of 10 nM Spire, addition of 20 nM FH1_D_-FH2 triggered fast elongation of over 90% filaments. Some of these filaments showed alternating periods of fast growth and arrested growth. 1 frame/10 s, 20-fold acceleration.
(AVI)

Movie S3 **Barbed end capping by Nt-Spire visualized in TIRF/microfluidics setup.** Filaments are elongating from surface-anchored spectrin-actin seeds in presence of 1 µM MgATP-G-actin, 4 µM profilin, and 5 nM Nt-Spire. Red arrows indicate capping events. Scale bar, 5 µm, 50-fold acceleration.
(AVI)

## Abbreviations

FH1: Formin Homology 1 domain
FH2: Formin Homology 2 domain
FYVE: Zinc finger domain named after the 4 cysteine rich proteins Fab1, YOTB, Vac1, and EEA1
KIND: Kinase Noncatalytic Domain
mDia1: mammalian Diaphanous 1
TIRF: Total Internal Reflection Fluorescence
WH2: WASP Homology 2 domain
