# Supplementary figures and images for: Spire and Formin 2 Synergize and Antagonize in Regulating Actin Assembly in Meiosis by a Ping-Pong Mechanism
Source: PLoS Biol. 2014 Feb 25;12(2):e1001795. doi: 10.1371/journal.pbio.1001795 (PMC3934834; doi:10.1371/journal.pbio.1001795)

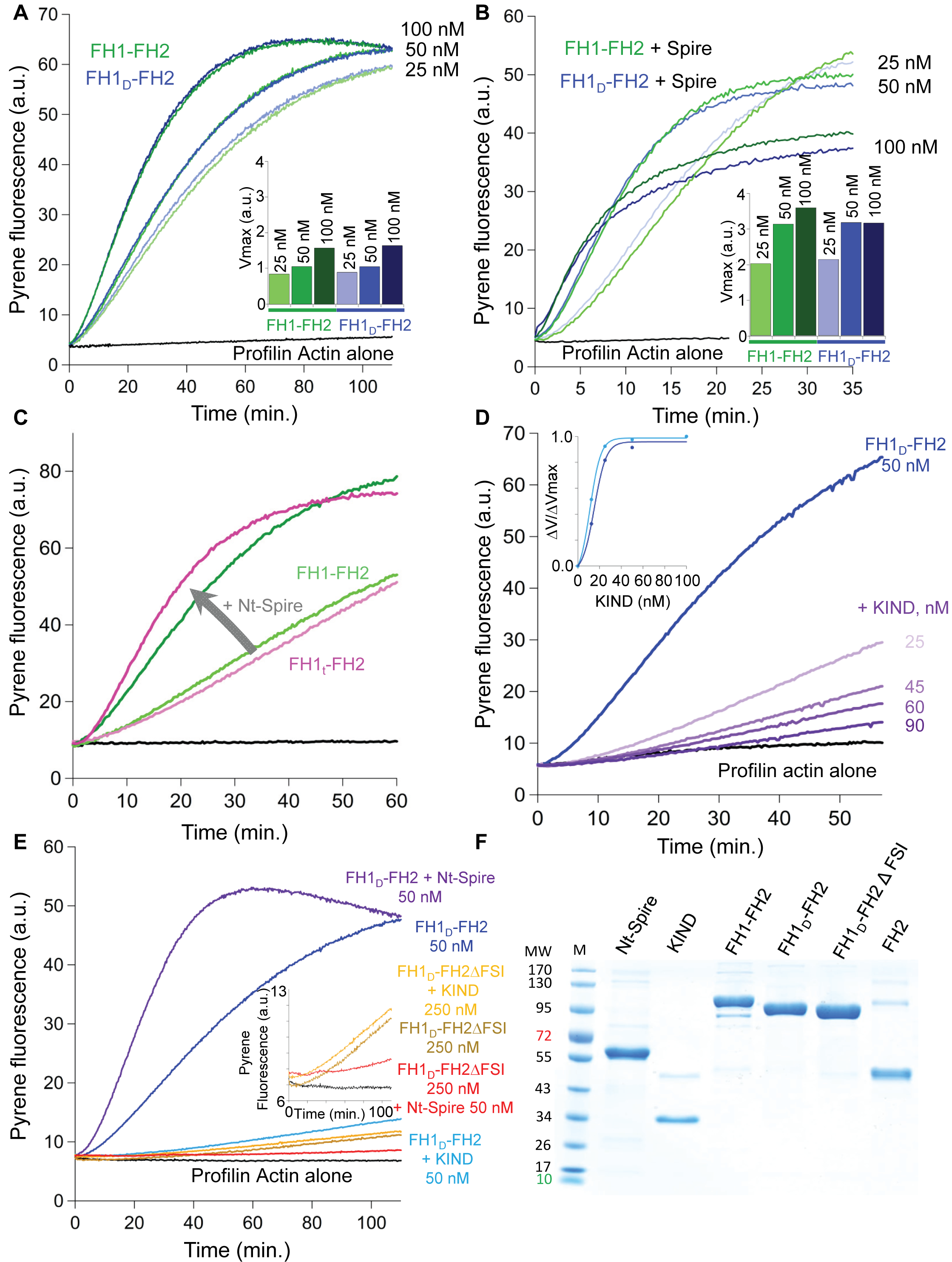

Supplement: Figure S1 — Validation of the FH1t-FH2 and FH1D-FH2 as substitutes of Fmn2 FH1-FH2. (A) FH1D-FH2 (blue lines) and original Fmn2 FH1-FH2 (green lines) display identical stimulation of actin assembly at a series of construct concentrations. (Inset) Histogram of the global asssembly rates, using same color coding as the raw data curves. (B) FH1D-FH2 (blue lines) and original Fmn2 FH1-FH2 (green lines) display identical synergy with Nt-Spire at a series of equimolar Nt-Spire∶FH1D-FH2 or Nt-Spire∶FH1-FH2 concentrations. (Inset) Histogram of the global asssembly rates, using same color coding as the raw data curves. (C) FH1t-FH2 (magenta lines) and original Fmn2 FH1-FH2 (green lines) display identical stimulation of actin assembly and functional interaction with Nt-Spire. (D) Dose dependence of the inhibition of FH1D-FH2 by KIND. (Inset) Maximal inhibition by KIND was reached at substoichiometric amount of KIND at 25 nM (light blue) and 50 nM Fmn2 (dark blue). (E) FH1D-FH2ΔFSI stimulates poorly filament assembly from profilin actin, is not affected by KIND, and is slightly inhibited by Nt-Spire. (Inset) The y-axis magnification of the actin assembly by FH1D-FH2ΔFSI, FH1D-FH2ΔFSI+KIND, and FH1D-FH2ΔFSI+Nt-Spire. All experiments in (A), (B), (C), (D), and (E) are with 2 µM actin, 4 µM profilin. (F) Coomassie Blue–stained SDS-PAGE of the various constructs used in the work. We loaded 30 pmoles of each protein in each lane. (TIF) [file pbio.1001795.s001.tif]

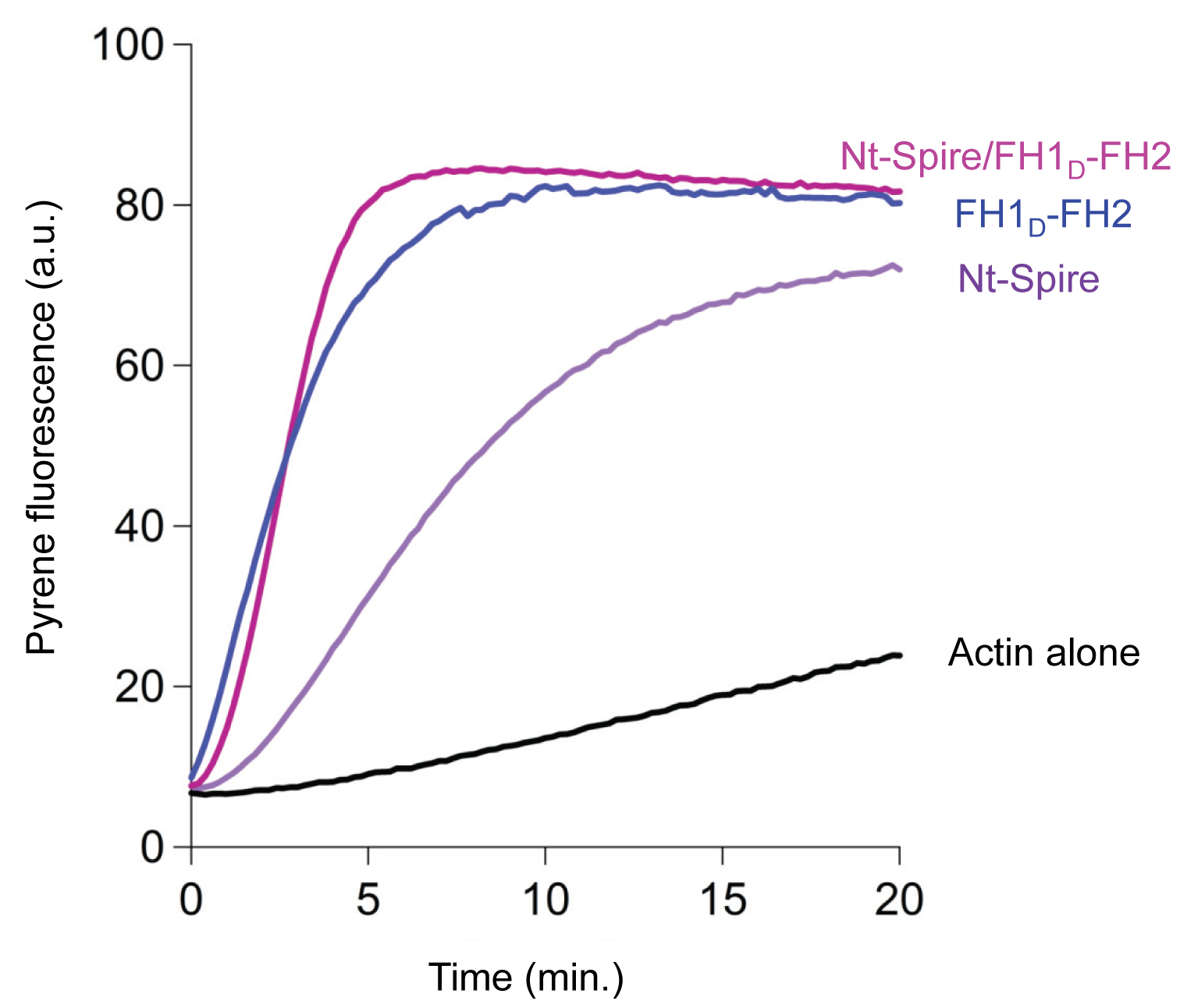

Supplement: Figure S2 — Stimulation of actin assembly by FH1D-FH2, Nt-Spire, and both proteins together in the absence of profilin. Conditions are 2.5 µM actin with or without 50 nM Nt-Spire or FH1D-FH2 or both proteins together. (TIF) [file pbio.1001795.s002.tif]

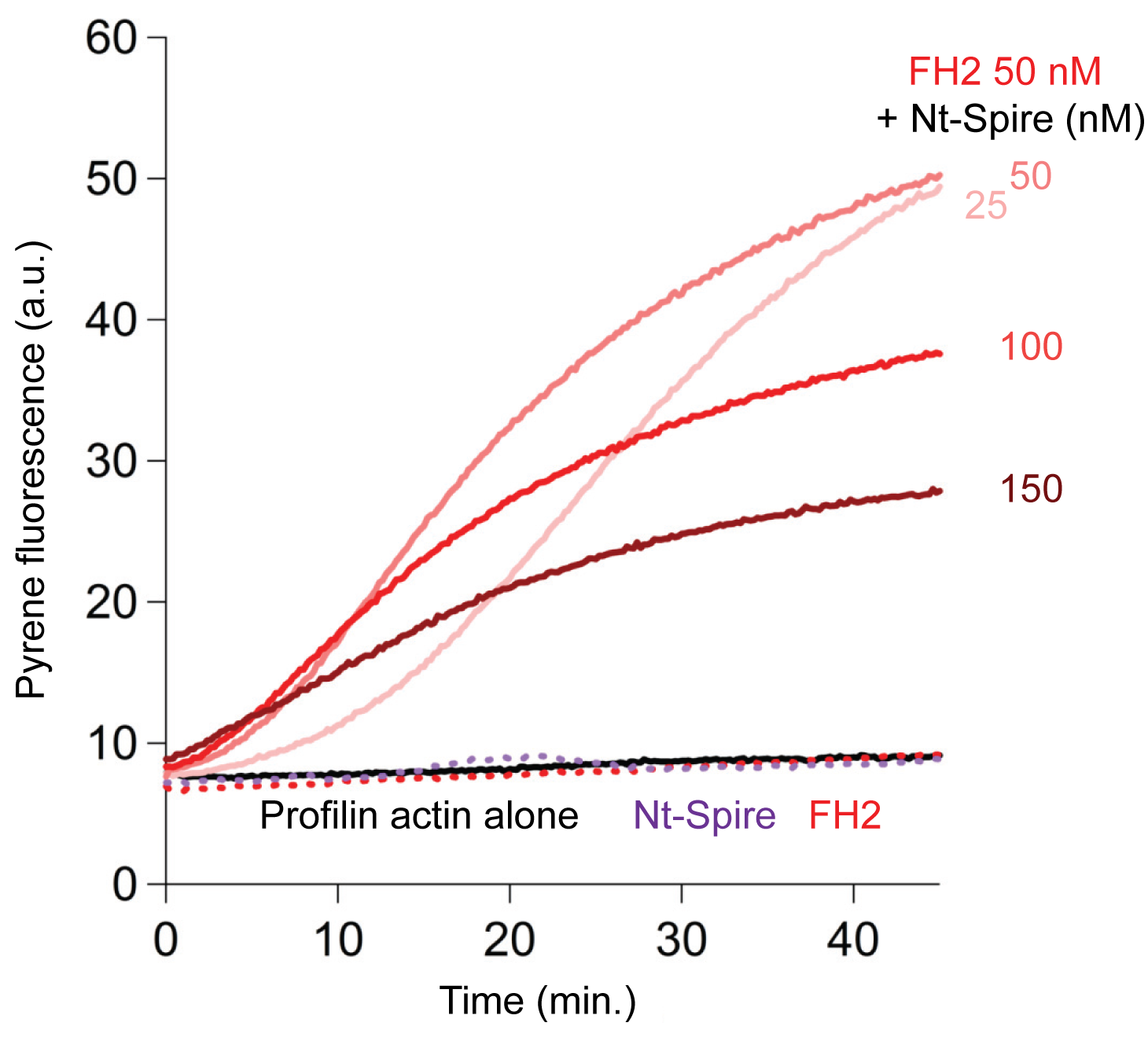

Supplement: Figure S3 — Spire induces filament assembly from PA in the presence of FH2. Actin (2.5 µM) was polymerized in the presence of 6 µM profilin (black line), 50 nM Spire (dotted purple line), and 50 nM FH2 without (red dotted line) or with increasing amounts of Spire (in nM). (TIF) [file pbio.1001795.s003.tif]

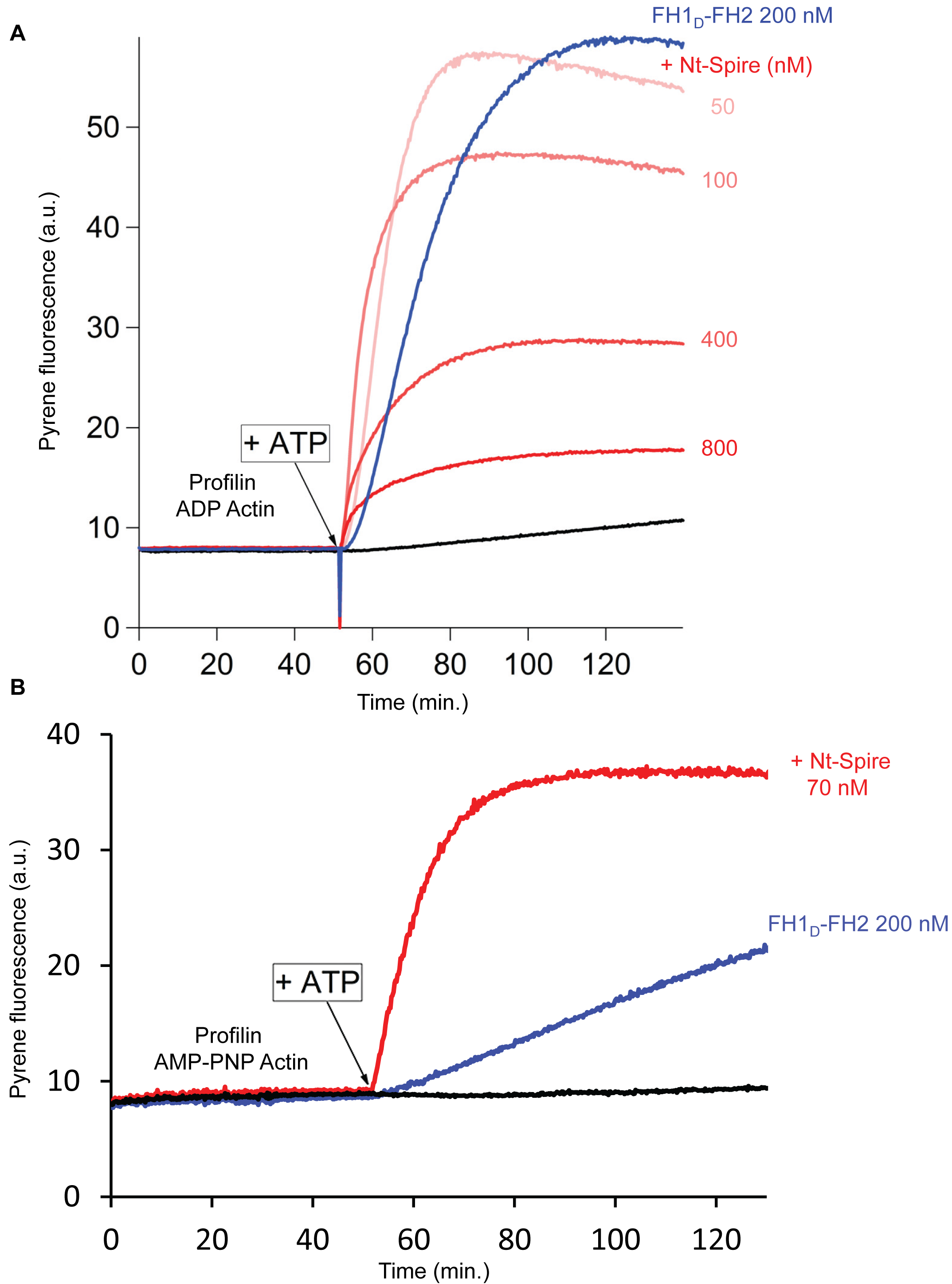

Supplement: Figure S4 — FH1D-FH2 fails to stimulate filament assembly from PA in the presence of ADP or AMPPNP. Actin was prepared in ADP- (A) or AMPPNP- (B) bound form (Materials and Methods) and assembled in the presence of profilin without (black lines) and with 200 nM FH1D-FH2, in absence (blue lines) or presence of Nt-Spire (red lines). ATP was then added (arrow). (TIF) [file pbio.1001795.s004.tif]

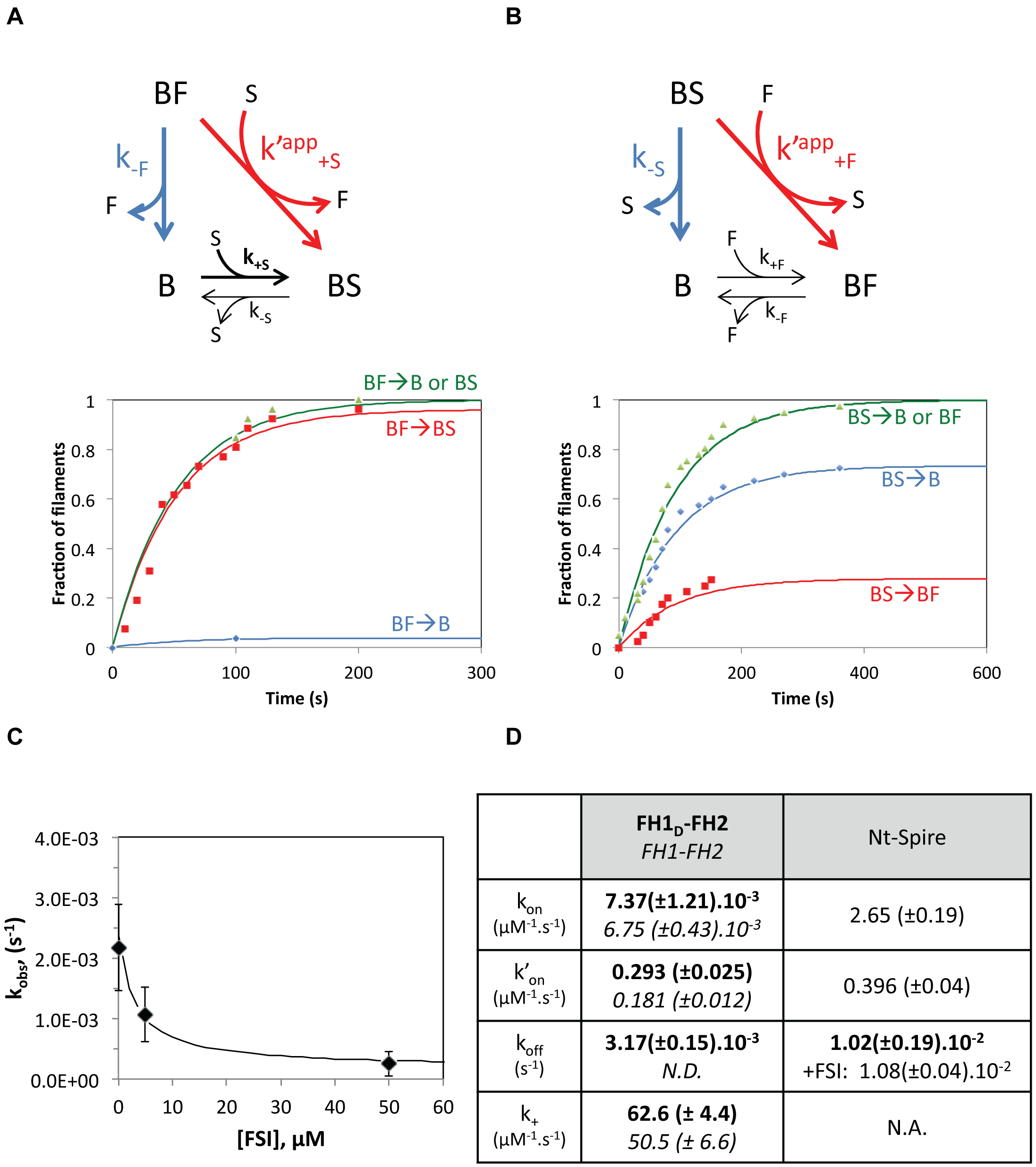

Supplement: Figure S5 — Measurement of the association rates of Spire (resp. Fmn2) to Fmn2- (resp. Spire-) bound barbed ends. (A) Fmn2-bound filament barbed ends (BF) are exposed to a flow of PA+Spire (without Fmn2). Arrest of rapid growth of Fmn2-bound barbed ends by Spire results from the combination of Fmn2 dissociation followed by association of Spire to a free barbed end and direct association of Spire to Fmn2-bound barbed ends, possibly followed by dissociation of Fmn2. The free barbed ends produced by the very slow reaction BF→B (rate constant k−F) grow slowly or are capped (transition from BF to BS). The reaction B→BS is rapid, and thus, the transition from state BF to BS includes the route BF→B→BS in addition to reaction BF→BS (with apparent rate constant k′app +S for a given concentration of Spire). Based on the resolution of our experiment and on the rate constants k+S and k−S, most filaments reaching state B are capped and convert to state BS very rapidly, while the vast majority (more than 90%) of the capped states BS last long enough to be identified unambiguously. Consistently, as shown at 40 nM Nt-Spire (graph), we observe almost only transitions from state BF to BS. The observed rate constant can be written kobs = k+S[S](k′app +S+k−F)/(k+S[S]+k−F). The data are fitted with k′app +S as a free parameter. The resulting k′app +S varies linearly with [Spire] (Figure 6D). (B) Spire-capped filaments (BS) are exposed to a flow of PA+Fmn2 (without Spire). Resumed fast growth at Spire precapped barbed ends results from two combined kinetic routes, dissociation of Spire followed by association of Fmn2 to a free barbed end, and direct association of Fmn2 to a Spire-capped barbed end possibly followed by rapid dissociation of Spire. Free barbed ends enter a state of slow growth (reaction BS→B, with rate constant k−s). Filaments that undergo reaction BS→BF, with the apparent rate constant k′app +F (for a given concentration of Fmn2), grow fast. The reactions B→BF and BF→B are slow, and [file pbio.1001795.s005.tif]

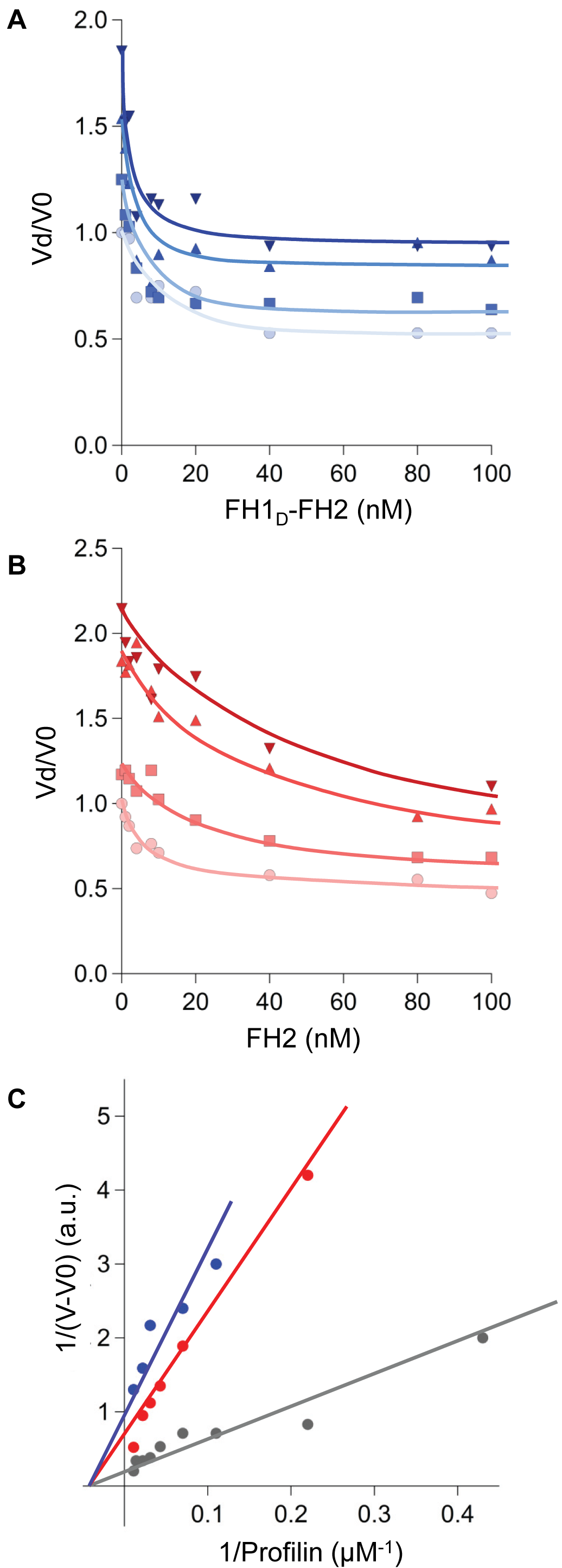

Supplement: Figure S6 — Profilin strengthens the binding of FH1D-FH2 at depolymerizing barbed ends. (A) Barbed end disassembly at the indicated concentrations of profilin is slowed down by FH1D-FH2 binding to barbed ends. The affinity of FH1D-FH2 for barbed ends is increased by profilin. (B) Barbed end disassembly at the indicated concentrations of profilin is slowed down by FH2. The affinity of FH2 for barbed ends is lowered by profilin. (C) Double reciprocal plots of the profilin concentration dependence of the rate of depolymerization from barbed ends in the absence (grey) and presence of either 100 nM FH2 (red) or FH1D-FH2 (blue). (TIF) [file pbio.1001795.s006.tif]
